# Loss of meningothelial identity and mesenchymal fate switching in NF2-mutant meningiomas

**DOI:** 10.1007/s00401-026-03016-3

**Published:** 2026-04-25

**Authors:** Ramin Rahmanzade, Leonille Schweizer, Simone Schmid, Tereza Lausová, Gianluca Sigismondo, Mozhgan Esmaeili Benvidi, Rushda Patel, Aras Fuat Kaan, Rouzbeh Banan, David Reuss, Natalie Jäger, Marie-Theresa Forster, Viktoria Zeller, Patricia Kohlhof-Meinecke, Annette Fisseler-Eckhoff, Barbara Carl, Patrick N. Harter, Katrin Lamszus, Ulrich Schüller, Michal Zapotocky, Till Acker, C. O. Hanemann, Jens Schittenhelm, Eva Brack, Uta Flucke, Gunhild Mechtersheimer, Luca Bertero, Christine Haberler, David Capper, Andreas von Deimling, Philipp Sievers, Felix Sahm

**Affiliations:** 1 Department of Neuropathology, Institute of Pathology, University Hospital Heidelberg, Heidelberg, Germany; 2 Present Address: Clinical Cooperation Unit Neuropathology (B300), German Cancer Research Center (DKFZ), Im Neuenheimer Feld 224, 69120 Heidelberg, Germany; 3 Institute of Pathology, Ludwig Maximilians University Hospital Munich, Munich, Germany; 4 Departments of Neuropathology, Edinger Institute, Institute of Neurology, University of Frankfurt Am Main, Heidelberg, Germany; 5 Frankfurt Cancer Institute (FCI), Frankfurt Am Main, Heidelberg, Germany; 6 German Cancer Consortium (DKTK), Partner Site Frankfurt/Mainz, German Cancer Research Center (DKFZ), Heidelberg, Germany; 7 Department of Neuropathology, Charité-Universitätsmedizin Berlin, Corporate Member of Freie Universität Berlin, Humboldt-Universität Zu Berlin, Berlin Institute of Health (BIH), Charitéplatz 1, 10117 Berlin, Germany; 8 German Cancer Consortium (DKTK), Partner Site Berlin, German Cancer Research Center (DKFZ), Heidelberg, Germany; 9 Institute of Pathology, Klinikum Stuttgart, Kriegsbergstrasse 60, 70174 Stuttgart, Germany; 10 Department of Pathology, Helios HSK Wiesbaden, 65199 Wiesbaden, Germany; 11 Department of Neurosurgery, University of Marburg, Marburg, Germany; 12 Department of Neurosurgery, Helios Dr. Horst Schmidt Kliniken, Wiesbaden, Germany; 13 Center for Neuropathology and Prion Research, LMU Munich, Munich, Germany; 14 Department of Neurosurgery, University Medical Center Hamburg-Eppendorf, 20246 Hamburg, Germany; 15 Institute of Neuropathology, University Medical Center Hamburg-Eppendorf, 20251 Hamburg, Germany; 16 Research Institute Children’s Cancer Center, 20251 Hamburg, Germany; 17 Department of Pediatric Hematology and Oncology, University Medical Center Hamburg-Eppendorf, 20251 Hamburg, Germany; 18 Second Faculty of Medicine, Prague Brain Tumor Research Group, Charles University and University Hospital Motol, V Uvalu 84, 15006 Prague 5, Czech Republic; 19 Pediatric Neurooncology Centre, University Hospital Motol, V Uvalu 84, 15006 Prague 5, Czech Republic; 20 Second Faculty of Medicine, Department of Pediatric Hematology and Oncology, Charles University Prague and University Hospital Motol, V Uvalu 84, 15006 Prague 5, Czech Republic; 21 Faculty of Health, Institute of Neuropathology, Justus Liebig University Giessen, Giessen, Germany; 22 Peninsula Medical School, University of Plymouth, PlymouthDevon, PL6 8BU UK; 23 Department of Neuropathology, University Hospital Tübingen, Eberhard-Karls-University Tübingen, Tübingen, Germany; 24 Division of Pediatric Hematology/Oncology, Department of Pediatrics, Inselspital, Bern University Hospital, University of Bern, Bern, Switzerland; 25 Department of Pathology, Radboud University Medical Center, Nijmegen, The Netherlands; 26 Diagnostic Laboratory, Princess Maxima Center for Pediatric Oncology, Utrecht, The Netherlands; 27 Institute of Pathology, University Hospital Heidelberg, Heidelberg, Germany; 28 Pathology Unit, Department of Laboratory Medicine, City of Health and Science University Hospital of Turin, Turin, Italy; 29 Pathology Unit, Department of Medical Sciences, University of Turin, Turin, Italy; 30 Division of Neuropathology and Neurochemistry, Department of Neurology, Medical University of Vienna, Vienna, Austria; 31 Hopp Children’s Cancer Center Heidelberg (KiTZ), Heidelberg, Germany

**Keywords:** Anaplastic meningioma, Sarcoma, NF2, SOX2, Lineage plasticity

## Abstract

Intracranial sarcomas can arise secondarily from primary brain tumors, including gliomas and meningiomas, either spontaneously or following radiotherapy. The current WHO classification recognizes sarcomatous transformation in several tumor entities; however, sarcomas arising from meningiomas remain poorly characterized and are regarded as a possible histological manifestation within the spectrum of anaplastic meningiomas. We analyzed nine matched meningioma–sarcoma pairs using integrated histopathological assessment and molecular profiling, including DNA methylation analysis, next-generation sequencing, copy number profiling, and proteomics. Although recurrent sarcomatous tumors were clonally related to their meningioma precursors—sharing identical NF2 alterations and overlapping chromosomal aberrations—they demonstrated pronounced divergence at the histological, immunophenotypic, and epigenetic levels. Importantly, sarcomatous transformation occurred in four cases without prior radiotherapy. Sarcomatous recurrences exhibited loss of meningothelial markers and acquired expression of cytokeratin and myogenic markers. DNA methylation profiling revealed a shift away from canonical meningioma signatures toward profiles resembling non-meningothelial mesenchymal tumors. Proteomic analysis showed consistent upregulation of SOX2 in sarcomatous tumors compared with their primary counterparts, suggesting acquisition of stem-like features during lineage divergence. Clinically, these tumors were associated with aggressive growth, early recurrence, and extracranial metastases, resembling malignant sarcomas more closely than anaplastic meningiomas. In addition, analysis of an institutional cohort of NF2-mutant intracranial tumors (n = 316) suggests that sarcomas with inactivating NF2 mutations may originate from meningiomas even in the absence of a clinically recognized precursor. Together, these findings suggest that sarcomatous transformation represents a rare evolutionary endpoint in NF2-mutant meningiomas, marked by clonal continuity but pronounced biological divergence. These results highlight limitations of morphology-based classification and emphasize the value of integrated molecular diagnostics in distinguishing these tumors from conventional high-grade meningiomas. Given their sarcoma-like behavior despite a meningioma ancestry, these tumors may not be adequately captured by current meningioma grading schemes.

**Supplementary Information:**

The online version contains supplementary material available at 10.1007/s00401-026-03016-3.

## Introduction

Intracranial sarcomas are rare entities that include primary, metastatic, and radiation-associated sarcomas [15, 22]. Additionally, sarcomatous transformation of primary brain tumors has been reported in a few entities, including glioblastoma [29], oligodendroglioma [11, 19], and meningioma [14, 18, 23]. However, sarcomatous transformation in meningiomas has been described primarily in isolated case reports and remains poorly characterized [28].

Previous reports have documented sarcoma-like morphology arising in recurrent meningiomas, occasionally following radiotherapy, and often associated with aggressive clinical behavior [14, 18, 23, 28]. Lucas et al. described three cases of sarcomatous meningiomas with myogenic differentiation, in which a meningioma origin was inferred based on focal expression of meningothelial markers and/or meningioma-typical molecular alterations, such as inactivating NF2 mutations or loss of chromosome 22q [23]. While these observations support a meningioma origin, the limited cohort size and absence of paired molecular analyses precluded systematic evaluation of clonal evolution, lineage plasticity, and biological distinction from anaplastic meningiomas.

From a diagnostic perspective, sarcomas arising from meningiomas pose a major challenge due to substantial morphological and immunohistochemical overlap with conventional soft tissue sarcomas. Accurate classification therefore requires integrated molecular approaches that can confirm clonal relatedness to a meningioma precursor while simultaneously delineating the extent of biological divergence.

In this study, we performed a comprehensive histopathological and multi-omics analysis of nine matched meningioma–sarcoma pairs. By combining histology, immunohistochemistry, DNA methylation profiling, copy number analysis, sequencing, and proteomics, we aimed to define the molecular trajectory of sarcomatous transformation in meningiomas and to clarify whether these tumors represent a biological endpoint distinct from conventional anaplastic meningiomas.

## Material and methods

### Tissue samples

A total of 179 recurrent intracranial tumors arising within or adjacent to the surgical cavity of a previously resected meningioma were identified during routine diagnostic evaluation at the Department of Neuropathology, University Hospital Heidelberg, between 2000 and 2024. All cases underwent comprehensive histopathological review by board-certified neuropathologists.

From this cohort, nine cases were selected based on the presence of predominantly sarcoma-like morphology in the recurrent tumor, raising significant diagnostic uncertainty with respect to anaplastic meningioma versus primary intracranial sarcoma. For each case, formalin-fixed paraffin-embedded (FFPE) tissue from the primary meningioma and the corresponding sarcomatous recurrence were available for molecular analyses.

Tissue collection, molecular analyses, and evaluation of clinical data were performed in accordance with local ethical regulations.

### Histology and immunohistochemistry

Histological evaluation was performed on hematoxylin–eosin- and reticulin-stained sections using standard protocols. Immunohistochemistry was carried out on a Ventana BenchMark ULTRA platform (Ventana Medical Systems, Tucson, AZ, USA).

The following antibodies were used: EMA (mouse monoclonal, clone E29, Ventana-Roche), SSTR2A (rabbit polyclonal, Biotrend), SMA (mouse monoclonal, clone HHF-35, Ventana-Roche), caldesmon (mouse monoclonal, clone hHCD, Medac), desmin (mouse monoclonal, clone D33, Dako), myogenin (rabbit monoclonal, clone EP162, Dako), Cytokeratin AE1/3 (mouse monoclonal, clone AE1/AE3, DCS), vimentin (mouse monoclonal, clone V9, Dako), GFAP (mouse monoclonal, clone GA5, Cell Signaling), Ki-67 (mouse monoclonal, clone MIB-1, Dako), SOX2 (rabbit polyclonal, Abcam), and INI-1 (mouse monoclonal, clone MRQ-27, Cell Marque).

### DNA methylation profiling and copy number analysis

DNA methylation profiling was performed on all primary and recurrent tumors (*n* = 18) using the Illumina Infinium HumanMethylation450 or MethylationEPIC BeadChip arrays, according to the manufacturer’s instructions and as previously described [5]. All computational analyses were performed using R version 4.6.1 (R Development Core Team 2020, https://www.R-project.org). *O6-methylguanine-DNA methyltransferase* (*MGMT*)-promoter methylation status was evaluated as previously described [5]. For unsupervised hierarchical clustering analysis of study cases and reference samples, the data were used as input for t-SNE analysis (t-distributed stochastic neighbor embedding). The following non-default parameters were applied: is_distance = T, theta = 0, pca = F, max_iter = 10,000, perplexity = 40. Tumor classification was explored using the Heidelberg Brain Tumor Classifier v12.8, the Sarcoma Classifier v13.1 and the Bethesda Classifier v2. These classifiers were applied to assess lineage relationships and epigenetic similarity rather than to establish definitive diagnostic assignments.

### RNA sequencing and fusion calling

RNA sequencing for the purpose of gene fusion calling was performed on a NextSeq 500 or NovaSeq 6000 instrument (Illumina) at the Department of Neuropathology Heidelberg as previously described [35].

### Next-generation DNA sequencing and mutational analysis

Next-generation DNA sequencing was performed for all recurrent tumors and their corresponding primary tumors (*n* = 18). All cases were sequenced at the Department of Neuropathology Heidelberg using a capture-based next-generation DNA sequencing approach on a NextSeq 500 or NovaSeq 6000 instrument (Illumina) applying a custom brain tumor panel as previously described [33].

### Mass spectrometry data acquisition

Proteins were extracted from FFPE punches of 1.5 mm in diameter with the four cycles of BeatBox-mediated (Preomics, Planegg/Martinsried, Germany) tissue shearing in SDS 4% lysis buffer, followed by SP3-based protein clean-up [17], and on-bead tryptic digestion (Promega, Fitchburg, WI, USA) in 50mM ammonium bicarbonate at 37 degrees for 16 h. The reaction was quenched with TFA 1% final, and 200 ng of peptides was separated on a 25 cm analytical column (75 μm x 250 mm, C18, 1.7 μm, 120 Å, Aurora 3, IonOpticks) using the nanoElute2 liquid chromatography system (Bruker Daltonics, Billerica, MA, USA) coupled to a trapped Ion mobility spectrometry time-of-flight HT mass spectrometer (Bruker Daltonics, Billerica, MA, USA) operated in positive mode (+ 1.6kV). Solvent A was water with 0.1% formic acid and solvent B was 80% acetonitrile and 0.1% formic acid. Peptides were separated over 120 min at 50°C. Peptides were acquired in data-independent acquisition mode with parallel accumulation—serial fragmentation (dia-PASEF) with a total cycle time of 1.7 s.

### Processing of proteomic data

Protein identification and quantification were conducted with DIA-NN (version 1.9) software [9] using default settings. Subsequent statistical analysis of whole proteome data was carried out in the programming language R version 4.3.1. Prior to imputation, protein intensities were normalized using the median Centering normalization implementation from the R-package “proBatch” [7]. In the following, the remaining missing values were imputed by using minDet imputation from the R-package “imputeLCMD”. Differential expression analysis was conducted via the R-package “limma” and Benjamini–Hochberg p-values [3]. Proteins of interest were defined as having an absolute log-fold change exceeding 1 and an adjusted P-value less than 0.05. Additional graphical representations if not stated otherwise were generated using “ggplot2” and “patchwork”.

## Results

### Copy number variation (CNV) and mutational analyses confirmed a clonal relationship between recurrent sarcomatous tumors and their corresponding primary meningiomas

A summary of demographic, radiologic, histomolecular, and clinical findings is provided in Table 1. All recurrent sarcomatous tumors (*n* = 9) and their respective primary counterparts (n = 9) underwent CNV and mutational profiling. CNV profiles were derived from DNA methylation data, while mutational analyses were performed using next-generation sequencing (NGS).

**Table 1:**
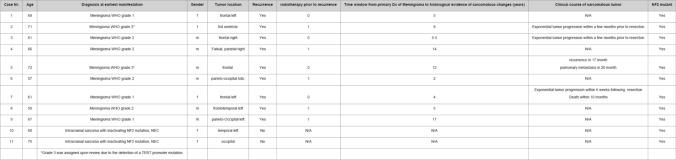
Summary of demographic and clinical findings

Shared CNV patterns between sarcomatous tumors and corresponding primary meningiomas were observed in all sarcoma–meningioma pairs except for Case #3, as summarized in Table 2 and Supplementary Fig. 1. Across the cohort, sarcomatous tumors most frequently exhibited losses of chromosomes 1p (6/9), 6q (4/9), 13q (6/9), and 22q (5/9), as well as gains of chromosomes 1q (4/9), seven (4/9), and eight (4/9). In addition, acquisition of a homozygous deletion of CDKN2A/B in the sarcomatous recurrence was observed in eight of nine cases.

**Table 2:**
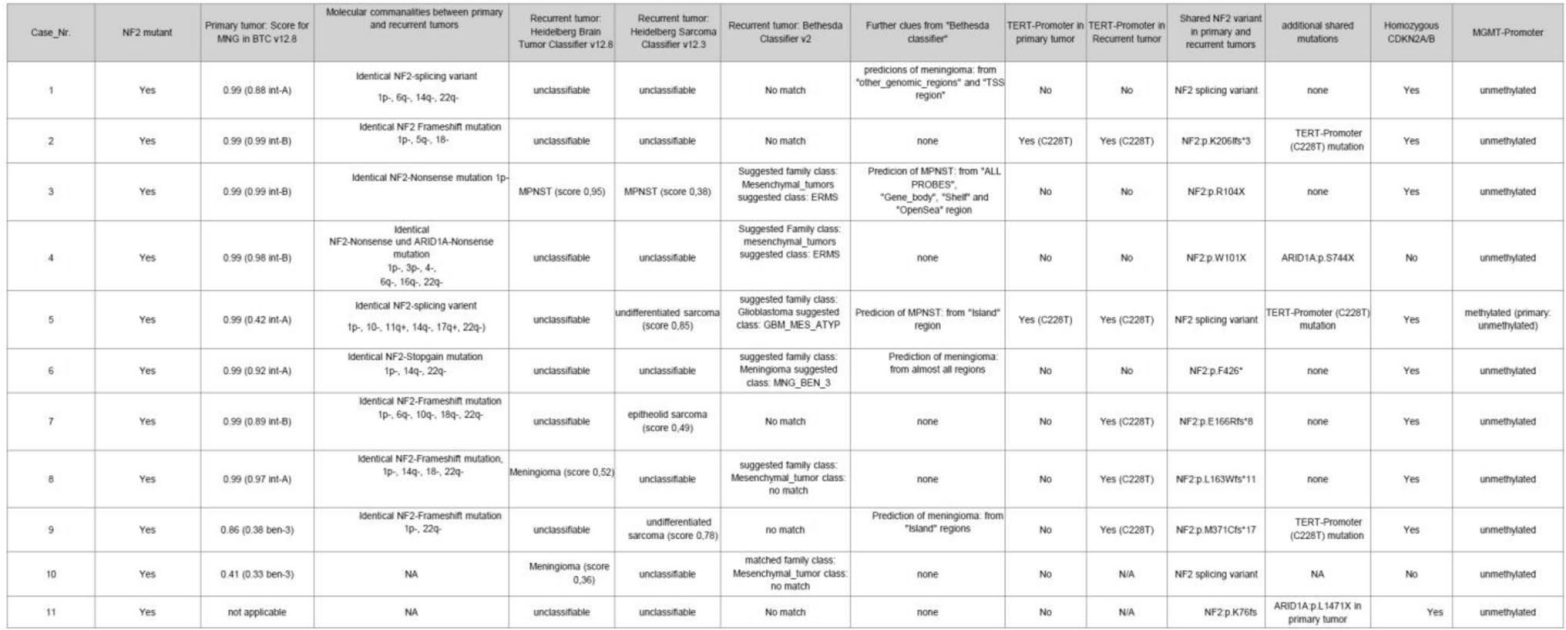
Summary of molecular findings

**Fig. 1:**
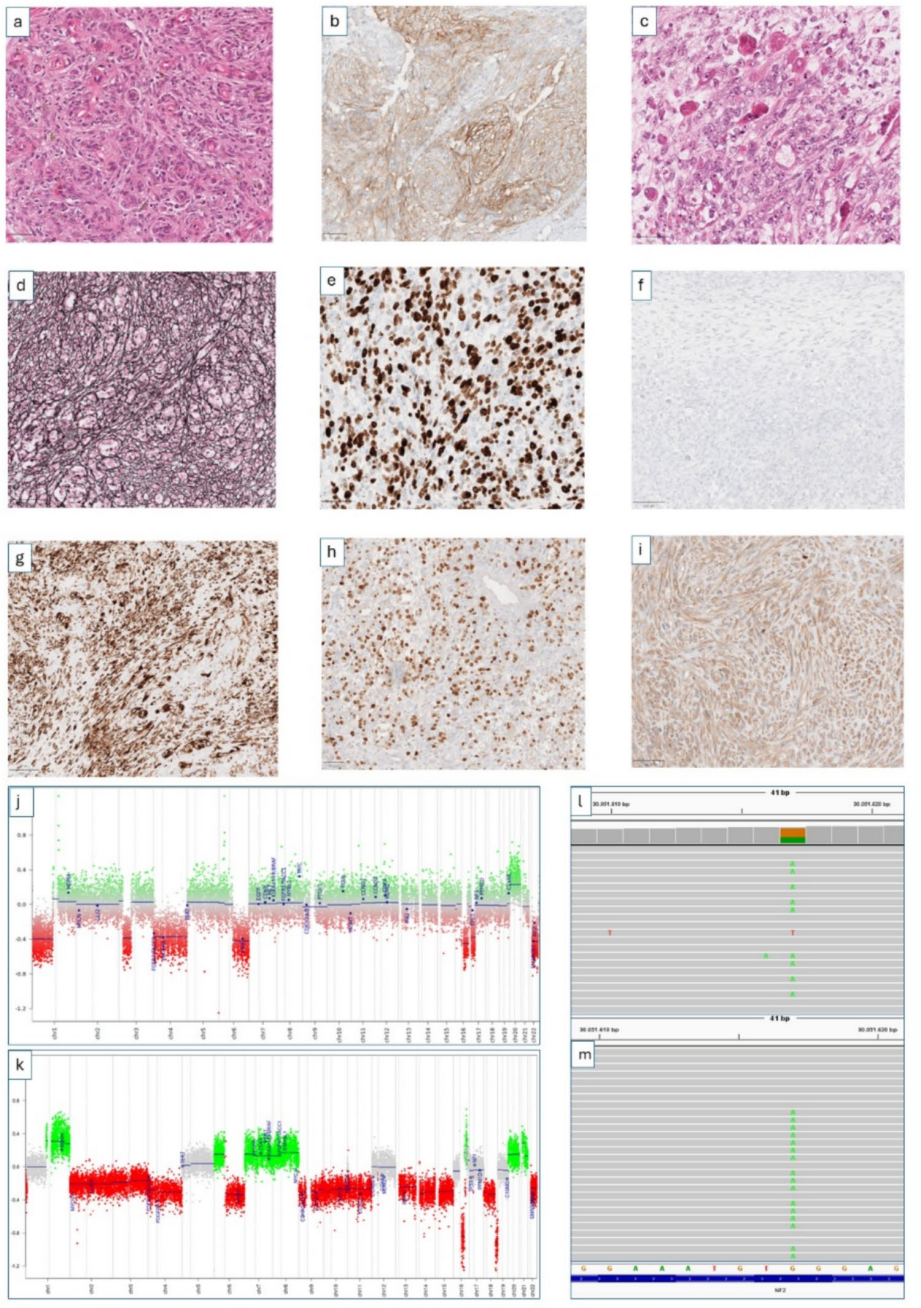
Sarcomatous transformation in a recurrent meningioma (#case 4). The primary tumor diagnosed in 2008 as a WHO grade 2 meningioma shows whorled architecture and nuclear pseudoinclusions **a **with SSTR2A positivity **b** and was classified as meningioma, int-B (score 0.97) using Brain Tumor Classifier v12.8. The recurrent tumor (2022) exhibits sarcomatous morphology with frequent round to oval cells with abundant cytoplasm implying myogenic differentiation (**c**), dense reticulin networks (**d**), high proliferative activity (**e**), and loss of meningothelial markers SSTR2A (**f**) and EMA (not shown). The sarcomatous tumor shows expression of myogenic markers desmin (**g**) and myogenin (**h**) as well as cytokeratin (**i**), whereas the primary tumor was negative for these markers (not shown). Copy number variation analysis demonstrates shared chromosomal losses in chromosomes 1p, 3p, 4, 6q, 16q, and 22q in both tumors (**j**–**k**). DNA sequencing identified identical NF2 nonsense and ARID1A nonsense mutations in the primary and recurrent tumors (**l**–**m**). t-SNE analysis based on DNA methylation profiles indicated closest proximity to embryonal rhabdomyosarcoma (see Fig. 4)

**Fig. 2:**
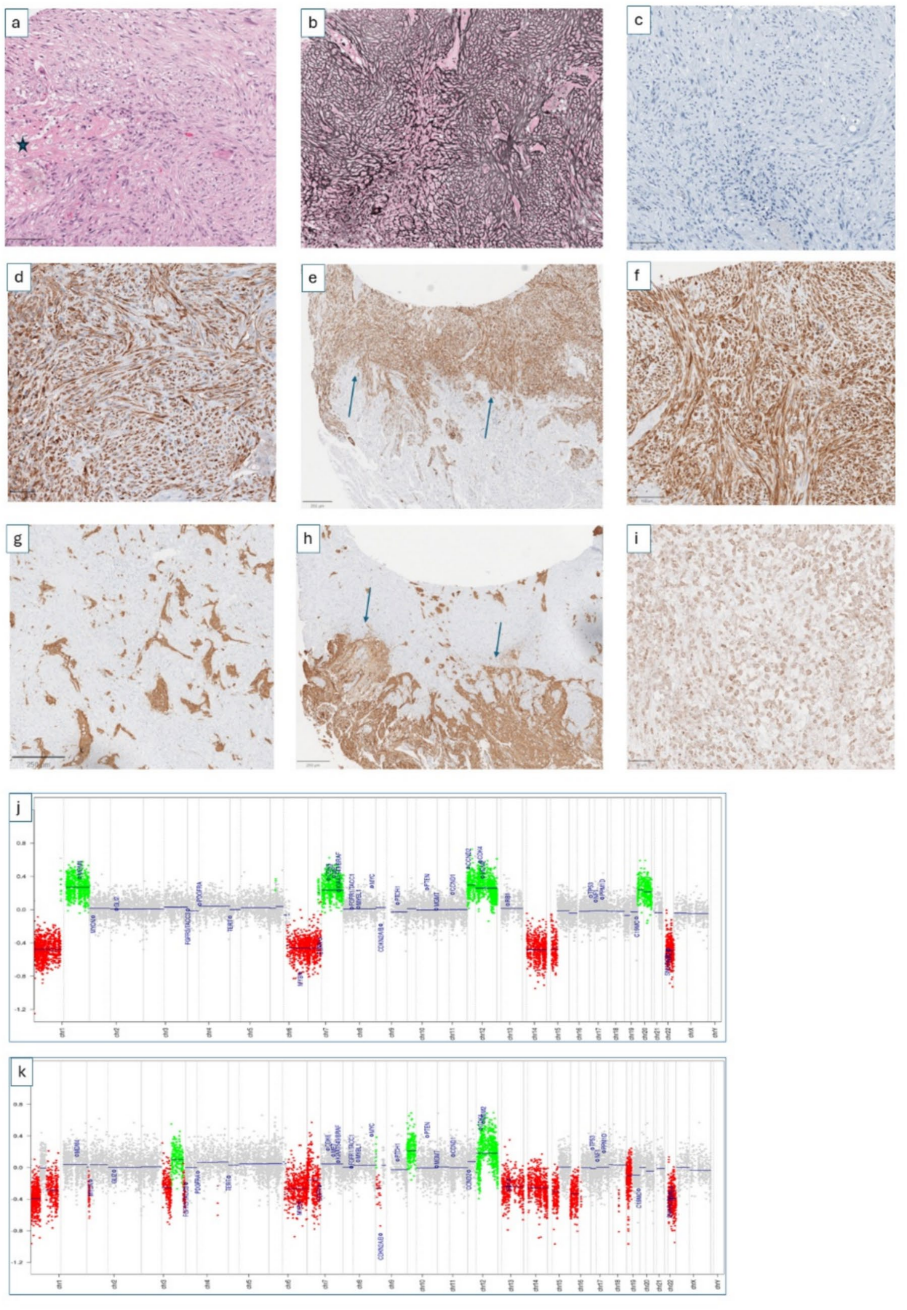
Sarcomatous recurrence of a WHO grade 2 meningioma (#case 1). The primary tumor diagnosed in 2004 showed meningothelial differentiation and was classified as MNG, int-A (score 0.90) using Brain Tumor Classifier v12.8. The recurrent tumor (2009) displayed sarcomatous morphology with long intersecting fascicles (**a**), areas of necrosis (a, asterisk), and dense reticulin networks (**b**). Tumor cells lost expression of meningothelial markers EMA (not shown) and SSTR2A (**c**) but showed strong positivity for SMA (**d**, **e**) and cytokeratin (**f**). Extensive invasion into adjacent CNS tissue was demonstrated by GFAP staining (**g**, **h**). Experimental immunohistochemistry showed nuclear SOX2 expression in tumor cells (**i**). Copy number analysis revealed shared chromosomal losses including 1p, 6q, 14q, and 22q in both tumors (**j**–**k**). DNA sequencing identified an identical NF2 splicing variant (22:30,032,739) in the primary and recurrent tumors. DNA methylation profiling was unclassifiable using Brain Tumor Classifier v12.8 and Bethesda classifier v2

NGS analysis identified identical NF2 gene alterations—including stop-gain mutations, frameshift deletions, and splice-site variants—in both sarcomatous tumors and their corresponding meningiomas in all cases (*n* = 9; see Figs. 1 and 2k,m). In Case #3, which showed limited overlap in CNV patterns, shared NF2 mutations were independently confirmed across multiple tumor regions by several NGS analyses (*n* = 4; data not shown). Although germline testing for NF2 was not available, there were no clinical features suggestive of neurofibromatosis type 2 syndrome.

Given the anatomical proximity of the recurrent sarcomatous tumors to their respective meningioma precursors and the presence of shared CNV patterns and identical NF2 alterations, a de novo occurrence of identical mutations in both tumors is highly unlikely. This interpretation is further supported by additional shared mutations beyond NF2, as detailed in Table 2. Specifically, *TERT* promoter mutations were identified in 5 of 9 cases, with newly acquired mutations detected in three recurrences (Case #7, #8 and #9). Moreover, an identical ARID1A nonsense mutation was present in both the primary and recurrent tumors in Case #4. RNA sequencing did not reveal any recurrent or diagnostically relevant gene fusions in the sarcomatous tumors.

**Fig. 3:**
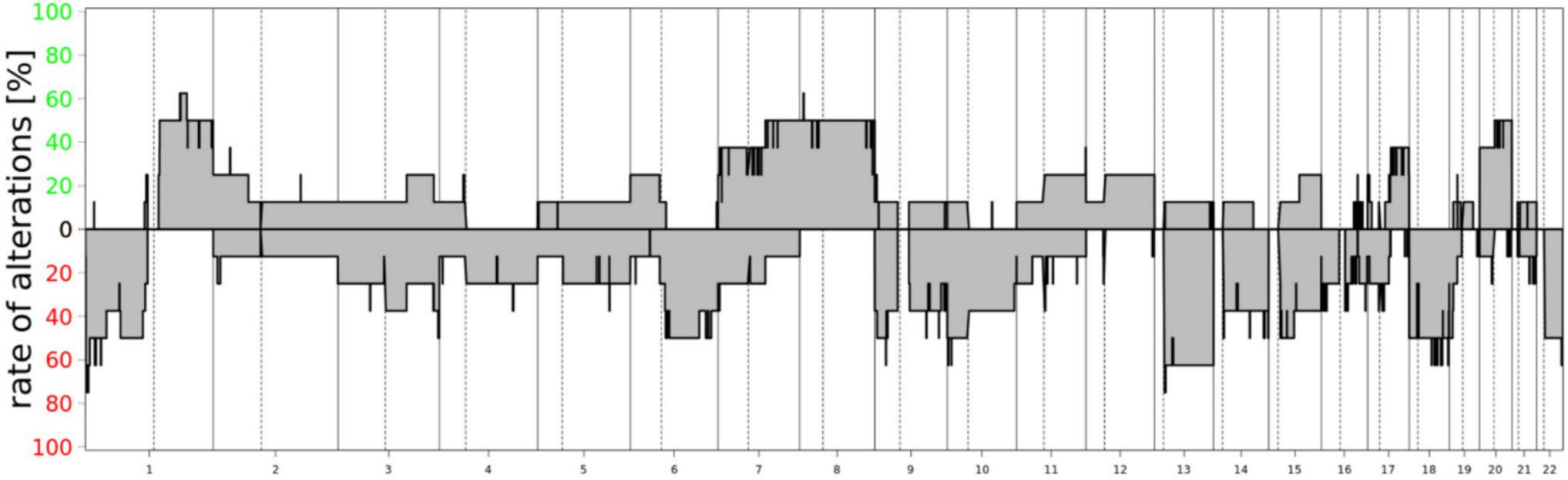
Cumulative copy number variation profile of recurrent sarcomatous tumors (*n* = 9) demonstrating frequent losses of chromosomes 1p, 6q, 13q, and 22q and gains of chromosomes 1q, 7, and 8. A focal loss of chromosome 9p including homozygous deletion of CDKN2A/B is observed in eight of nine cases

The cumulative CNV profile of the recurrent sarcomatous tumors is shown in Fig. 3.

### Histological and immunohistochemical analyses demonstrate loss of meningothelial differentiation and acquisition of myogenic features in sarcomatous recurrences

All recurrent sarcomatous tumors (n = 9) and their corresponding primary meningiomas (*n* = 9) underwent comprehensive histological and immunohistochemical evaluation, as summarized in Table 3. Histologically, all sarcomatous recurrences displayed a predominantly sarcoma-like growth pattern, characterized either by sheets of epithelioid cells or by storiform and interlacing fascicles of spindle cells. Frequent mitotic figures and areas of tumor necrosis were observed in all cases. Dense pericellular reticulin networks were present throughout the sarcomatous components in all tumors.

**Table 3:**
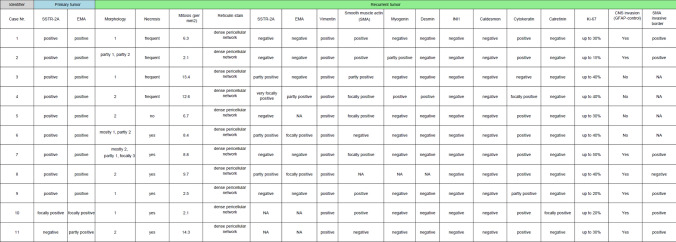
Summary of histological and immunohistochemical findings

Histological patterns were classified as follows: ^1^ storiform pattern of spindle cells, ^2^ sheeting architecture of epithelioid cells, ^3^ meningothelial differentiation

In five cases, adjacent CNS tissue was included in the resection specimen, allowing evaluation of the tumor–brain interface. In all of these cases (Cases #1, #3, #4, #7, and #9), GFAP immunohistochemistry demonstrated extensive invasion of tumor cells into the surrounding brain parenchyma (Fig. 2g–h; Supplementary Figs. 2 and 3).

The primary tumors were histologically classified as meningiomas of transitional subtype (*n* = 5), fibroblastic subtype (*n* = 2), meningothelial subtype (*n* = 1), and one tumor with sheeting architecture. They have been graded according to the WHO classification of brain tumors into grade 3 (*n* = 2), grade 2 (*n* = 4), and grade 1 (*n* = 2). Of note, detection of TERT promoter mutations led to upgrading of two primary meningiomas initially diagnosed as WHO grade 2 to WHO grade 3 (Cases #2 and #5). All primary meningiomas showed uniform expression of EMA and SSTR2A. In contrast, the sarcomatous recurrences showed complete loss of EMA and SSTR2A expression in five cases, while four cases retained only focal or patchy positivity for one or both markers. Notably, none of the primary meningiomas showed expression of myogenic markers.

Conversely, sarcomatous recurrences demonstrated prominent expression of non-meningothelial markers. α-Smooth muscle actin (αSMA) was expressed in seven of nine cases, cytokeratin AE1/3 in eight of nine cases, and vimentin in all cases (9/9). Expression of myogenic markers was observed in a subset of tumors, with myogenin positivity in two cases and desmin positivity in one case. Notably, expression of αSMA and cytokeratin was often most pronounced at the tumor–brain interface (Figs. 1 and 2; Supplementary Figs. 3 and 4).

Importantly, none of the corresponding primary meningiomas exhibited even focal expression of cytokeratin, αSMA, desmin, or myogenin. This distinct immunophenotypic shift was consistently observed across all matched tumor pairs.

### DNA methylation profiling reveals epigenetic divergence of sarcomatous recurrences toward non-meningothelial mesenchymal lineages

DNA methylation profiling was performed on all primary meningiomas (*n* = 9) and their corresponding sarcomatous recurrences (*n* = 9). Results are summarized in Table 2.

Using the Brain Tumor Classifier v12.8, all primary tumors were classified as meningiomas with high confidence. In contrast, the majority of sarcomatous recurrences were unclassifiable within the brain tumor reference set. One sarcomatous recurrence (Case #3) was classified as malignant peripheral nerve sheath tumor (MPNST). In Case #8, the sarcomatous recurrence achieved the highest classifier score for meningioma despite sarcoma-like histology and immunophenotype.

Application of the Sarcoma Classifier v13.1, as recently described by Jäger et al. (2025, preprint), identified two sarcomatous recurrences (Case #5 and Case #9; shown in Supplementary Fig. 4) with high classification scores for undifferentiated sarcoma. The remaining sarcomatous tumors did not reach high-confidence classification but showed highest similarity to sarcoma entities, including undifferentiated sarcoma (*n* = 4), epithelioid sarcoma (Case #7), and MPNST (Case #3).

To further explore lineage relationships, all tumors were additionally analyzed using the Bethesda Classifier v2, which applies multiple independent classifiers targeting distinct genomic regions [28]. With this approach, all primary tumors were again classified as meningiomas. In addition, the sarcomatous recurrence in Case #6 was classified as a meningioma. In Cases #1 and #9, methylation signatures from selected genomic regions showed similarity to meningioma reference classes (Table 2; Supplementary Fig. 5).

To assess global epigenetic relationships, an unsupervised* t*-distributed stochastic neighbor embedding (*t*-SNE) analysis was performed on a dataset comprising 228 samples, including reference sarcomas from the Heidelberg Sarcoma Classifier training set (*n* = 150), the sarcomatous recurrences (*n* = 9) and their corresponding primary meningiomas (*n* = 9), recurrent non-sarcomatous meningiomas with their primaries (*n* = 20), glioblastomas (*n* = 10), gliosarcomas (*n* = 10), oligodendrogliomas (*n* = 10), and oligosarcomas (*n* = 10).

**Fig. 4:**
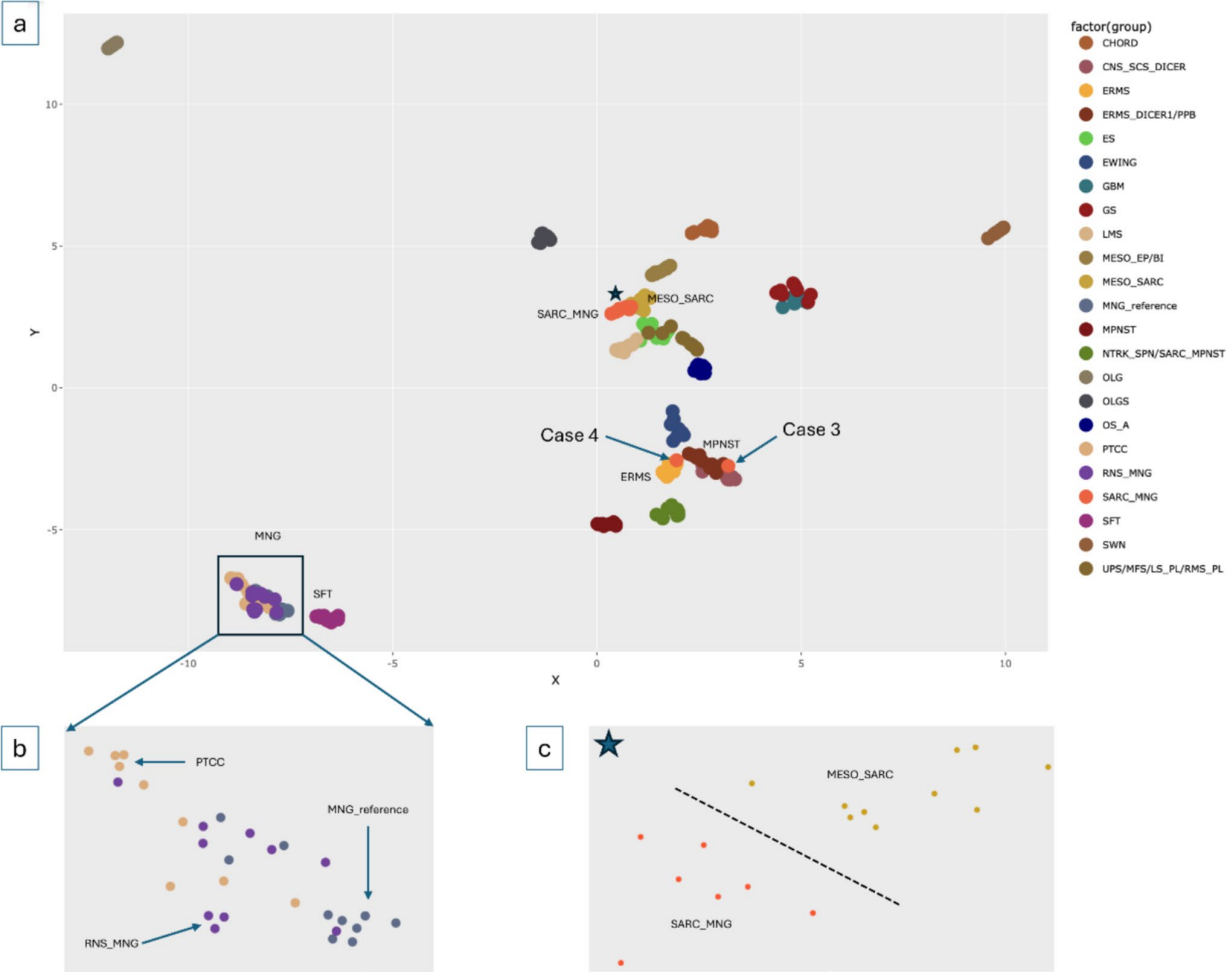
Unsupervised, non-linear t-distributed stochastic neighbor embedding (t-SNE) projection of DNA methylation array profiles from 228 samples. The analysis includes reference cohorts for various sarcomas (*n* = 150), cohort cases with sarcomatous transformation (*n* = 9) and their primary meningioma counterparts (*n* = 9), recurrent non-sarcomatous meningiomas (*n* = 10) with their corresponding primary meningiomas (*n* = 10), as well as glioblastomas (*n* = 10), gliosarcomas (*n* = 10), oligodendrogliomas (*n* = 10), and oligosarcomas (*n* = 10). Diagnoses of all included cases were verified by thorough histological and molecular review by board-certified pathologists and neuropathologists. Recurrent non-sarcomatous meningiomas and their primary counterparts clustered together with primary meningiomas of the cohort cases within the meningioma reference class (**b**). While gliosarcomas showed the closest epigenetic resemblance to glioblastomas, recurrent sarcomatous tumors from the study cohort largely diverged from meningiomas and displayed epigenetic proximity to non-meningioma sarcoma classes (**a**). A subset of recurrent sarcomatous tumors (*n* = 7) clustered together (asterisk in a), located adjacent to but distinct from the reference class of mesothelial sarcoma (**c**). Consistent with its histological features, case 4 clustered with embryonal rhabdomyosarcomas, whereas case 3, classified as malignant peripheral nerve sheath tumor by Brain Tumor Classifier v12.8, localized near malignant peripheral nerve sheath tumor and DICER1-associated embryonal rhabdomyosarcoma reference samples. Abbreviations: *EWING* Ewing sarcoma, *SFT* solitary fibrous tumor, *LMS* leiomyosarcoma, *ERMS* embryonal rhabdomyosarcoma, *SWN* schwannoma, *MPNST* malignant peripheral nerve sheath tumor, *NTRK_SPN/SARC_MPNST* MPNST-like Sarcoma, *MESO_SARC* mesothelial sarcoma, *MESO_EP/BI* epithelioid mesothelioma, *ES* epithelioid sarcoma, *UPS/MFS/LS_PL/RMS_PL* undifferentiated sarcoma, *OS_A* high-grade osteosarcoma, *CHORD* chordoma, *CNS_SCS_DICER* Primary CNS sarcoma with DICER1 mutation, *SARC_MNG* sarcomatous meningioma (cohort cases), *PTCC* primary tumor counterparts of cohort cases, *RNS_MNG* recurrent non-sarcomatous meningioma, *GBM* glioblastoma, *GS* gliosarcoma, *OLG* oligodendroglioma, *OLGS* oligosarcoma

Recurrent non-sarcomatous meningiomas included WHO grade 3 (*n* = 6) and grade 2 (*n* = 4) tumors arising from primary grade 1 or grade 2 meningiomas. This reference set encompassed tumors with diverse histological grades and clinical backgrounds, including cases with prior radiotherapy. In this analysis, primary meningiomas from the cohort clustered with reference meningiomas and with recurrent non-sarcomatous meningiomas and their primaries (Fig. 4b). In contrast, sarcomatous recurrences showed marked epigenetic divergence and aligned with a broad range of non-meningothelial mesenchymal tumor classes, including embryonal rhabdomyosarcoma (ERMS), MPNST, DICER1-associated embryonal rhabdomyosarcoma, and mesothelial sarcoma (Fig. 4a).

A subset of sarcomatous recurrences (*n* = 7) formed a distinct cluster (Fig. 4a, marked with *) located adjacent to, but separate from, the reference cluster of sarcomatous mesotheliomas (Fig. 4c). Consistent with histological and classifier-based findings, Case #4 clustered with ERMS, while Case #3 (Supplementary Fig. 2) localized in the proximity of the MPNST cluster.

Extended t-SNE analyses incorporating the combined reference datasets of the Heidelberg Brain Tumor Classifier v12.8 and the Sarcoma Classifier v13.1, which include a broader range of tumor entities, yielded comparable results and further supported the epigenetic divergence of sarcomatous recurrences from their meningioma precursors, with distinct clustering observed for seven of nine cases.

### Proteomic profiling identifies consistent SOX2 upregulation and DMGDH downregulation in sarcomatous recurrences

**Fig. 5:**
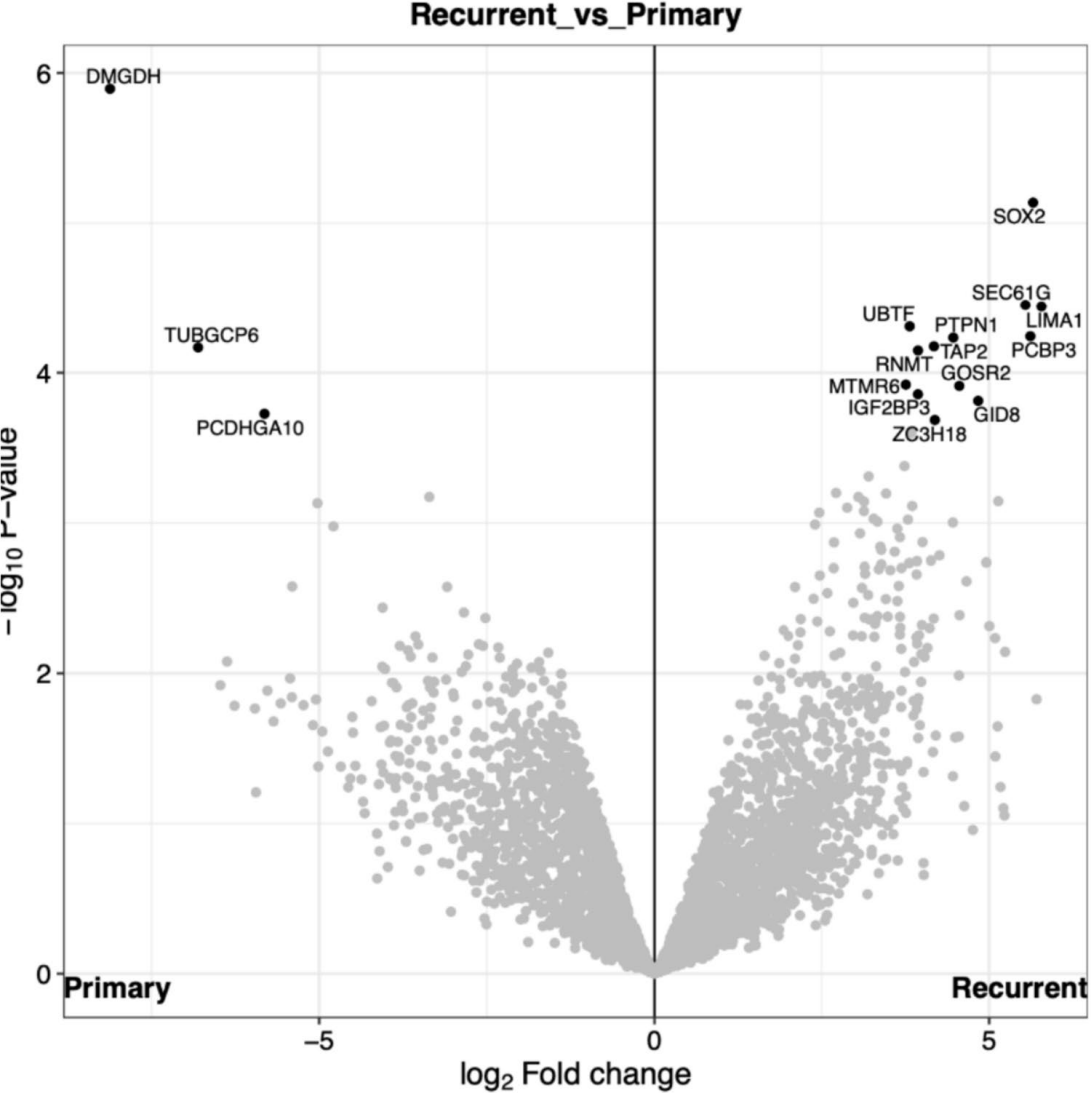
Volcano plot of differentially abundant proteins comparing recurrent sarcomatous tumors with their primary meningioma counterparts. SOX2 is among the most significantly upregulated proteins, whereas dimethylglycine dehydrogenase (DMGDH) is markedly downregulated in sarcomatous tumors

Comparative proteomic analysis of recurrent sarcomatous tumors and corresponding primary meningiomas revealed significant differences in protein expression levels, as visualized in a volcano plot (Fig. 5). Differential expression analysis identified SOX2 as one of the most significantly upregulated proteins in recurrent sarcomatous tumors compared with primary meningiomas, showing a log₂-fold change greater than 2 and a high level of statistical significance. In contrast, DMGDH (dimethylglycine dehydrogenase) was among the most significantly downregulated protein in recurrent tumors relative to their primary counterparts. Notably, there was a consistent pattern of SOX2 upregulation and DMGDH downregulation upon recurrence.

In addition to SOX2 and DMGDH, several other proteins showed statistically significant differential expressions between recurrent and primary tumors, as indicated in Fig. 5.

### Intracranial sarcoma with inactivating NF2 mutation, not elsewhere classifiable (NEC): arising from meningiomas even if no prior meningioma is known?

#### Case-based analyses

In addition to the nine meningioma–sarcoma pairs, two primary intracranial tumors with sarcomatous morphology and inactivating NF2 mutations were identified during routine diagnostic workup between 2000 and 2024 (Case #10 and Case #11). Neither case had a documented prior diagnosis of meningioma.

Histologically, both tumors showed predominantly sarcomatous morphology. Immunohistochemically, focal expression of meningothelial markers, including EMA and SSTR2A, was observed in both cases, alongside prominent expression of mesenchymal markers, including α-smooth muscle actin (αSMA), cytokeratin, and vimentin.

In Case #10, intraoperative findings documented direct contact of the tumor with the meninges. DNA methylation profiling was performed separately on SSTR2A-positive and SSTR2A-negative tumor regions. Both regions showed the highest similarity to meningioma reference classes and achieved the highest classification scores for meningioma (MNG) using the Heidelberg Brain Tumor Classifier v12.8 (scores 0.41 and 0.36, respectively). Consistent with these results, Case #10 clustered with meningiomas in a UMAP analysis incorporating the reference dataset used for training the Bethesda classifier.

Both tumors shared molecular and histopathological features observed in sarcomatous recurrences from the paired cohort. In addition to the inactivating NF2 mutation, Case #10 demonstrated extensive invasion into adjacent CNS tissue (Supplementary Fig. 5d). Notably, this case localized within the cluster of meningiomas in the t-SNE analysis (Fig. 4). Case #11 exhibited loss of chromosome 22q, homozygous deletion of CDKN2A/B, gains of chromosomes 7 and 8, and a nonsense mutation in ARID1A. Furthermore, this case clustered within the group of sarcomatous tumors arising from meningiomas in the t-SNE analysis (Fig. 4), supporting a possible origin from a meningioma precursor.

### Distribution of intracranial NF2-mutant tumors

A total of 316 intracranial tumors with pathogenic or likely pathogenic NF2 mutations were identified in the institutional database of the Institute of Neuropathology, University Hospital Heidelberg. Following histological and molecular review, 292 cases with sufficient material received a definitive diagnosis, including 255 cases in which the diagnosis was corroborated by DNA methylation analysis.

The majority of tumors were classified as meningiomas (*n* = 261). Additional diagnoses included sarcomas with inactivating NF2 mutations, not elsewhere classifiable (NEC) (*n* = 11), schwannomas (*n* = 8), high-grade gliomas (*n* = 5), ependymomas (*n* = 3), metastases (*n* = 3), and epithelioid malignant peripheral nerve sheath tumor (MPNST) (*n* = 1).

Sarcomas with inactivating NF2 mutations identified in the database were limited to the tumors described in this study (Cases #1–#11).

## Discussion

Intracranial soft tissue sarcomas carrying inactivating NF2 mutations are exceptionally rare, with only isolated cases reported to date [23, 26]. These reports highlight the diagnostic challenge posed by intracranial sarcomas with NF2 alterations, particularly when no antecedent meningioma is clinically recognized. In this context, our study provides the most comprehensive molecular characterization to date of sarcomas arising from meningiomas, selected from a large cohort of recurrent meningiomas, and establishes a clear clonal relationship between meningiomas and their sarcomatous counterparts based on shared genetic alterations and overlapping copy number profiles.

Despite clonal relatedness, sarcomatous tumors showed profound phenotypic and epigenetic divergence from their precursor meningiomas. Immunohistochemical and DNA methylation analyses demonstrated loss of meningothelial differentiation and acquisition of heterogeneous mesenchymal phenotypes, in contrast to glioblastoma-derived sarcomas, which typically retain close epigenetic proximity to their glial counterparts. These findings indicate a high degree of lineage plasticity in meningiomas undergoing sarcomatous transformation.

Proteomic profiling further supported this concept by identifying consistent upregulation of SOX2 and downregulation of DMGDH in sarcomatous tumors compared with primary meningiomas. SOX2 has previously been associated with high-grade meningiomas and adverse clinical outcome [1, 10] and is linked to stem-like or progenitor-like tumor cell states. While our data do not establish a functional role for SOX2, its consistent overexpression is compatible with acquisition of cellular plasticity during tumor progression. Reduced expression of DMGDH, previously described as a metastasis-suppressor-associated gene in other malignancies [21], may reflect metabolic or differentiation changes accompanying anaplastic progression.

Our proteomic analysis was not designed to systematically compare marker expression across different meningioma subtypes or sarcoma entities. Nevertheless, the consistent upregulation of SOX2 may reflect acquisition of stem-like cellular states during lineage transition. While these findings primarily suggest a biological role of SOX2 in the observed lineage plasticity, its expression may also provide supportive diagnostic information in the context of sarcomatous transformation.

Importantly, sarcomatous transformation in our cohort was restricted to tumors harboring inactivating NF2 alterations. This consistent association suggests that an NF2-mutant background in meningiomas may provide a permissive genomic context for phenotypic instability and lineage deviation toward non-meningothelial mesenchymal states. In line with this observation, pathogenic NF2 mutations in meningiomas have previously been associated with higher tumor grade, chromosomal instability, early recurrence, and adverse clinical outcome [16, 27, 32].

The link between NF2 loss and altered cellular differentiation in meningiomas has been recognized for decades. Early studies demonstrated that NF2-mutant meningiomas more frequently exhibit fibroblastic or spindle-cell morphology compared with meningothelial subtypes, indicating a shift toward mesenchymal-like differentiation already at the level of conventional meningiomas [37]. Mechanistically, loss of Merlin/NF2 has been linked to deregulation of Hippo–YAP/TAZ signaling and associated transcriptional programs that promote phenotypic plasticity and heterogeneous cell-state programs in NF2-mutant tumors [6, 20, 24, 38]. Within this framework, sarcomatous transformation may represent an extreme evolutionary end point along a broader differentiation spectrum in NF2-mutant meningiomas, rather than a separate biological process.

Epigenetic clustering analysis further identified a distinct subset of tumors that grouped together (Fig. 4), indicating shared epigenetic features that were distinct from both conventional meningiomas and fully differentiated sarcomas. These observations are compatible with the presence of heterogeneous or partially divergent cellular states during lineage reprogramming and sarcomatous evolution [12, 13, 24, 30, 31, 34].

Historically, inconsistent terminology—particularly the use of meningosarcoma—has contributed to confusion in this field [2, 4, 8, 14, 18, 25, 29]. In our cohort, sarcomas arising from meningiomas were associated with an aggressive clinical course, including rapid growth, early recurrence and, in rare cases, extracranial metastasis—features that may exceed what is typically observed in conventional anaplastic meningiomas [36, 37]. Importantly, these tumors also showed marked immunophenotypic and epigenetic differences from conventional anaplastic meningiomas, with some cases displaying greater similarity to soft tissue sarcomas than to meningiomas. While we do not propose a distinct tumor entity, our findings suggest that sarcomatous transformation represents an uncommon evolutionary trajectory within NF2-mutant meningiomas that may not be fully captured by morphology-based classification alone. In this context, the historical term *meningosarcoma* may still be used descriptively for sarcomas arising from meningiomas, particularly in cases in which a meningioma precursor can be identified.

In summary, our findings demonstrate that sarcomatous transformation represents a rare but distinct evolutionary trajectory within NF2-mutant meningiomas, characterized by clonal continuity with profound molecular and phenotypic divergence. Integrated molecular diagnostics are essential for accurate classification of these tumors and for distinguishing them from primary intracranial sarcomas. Larger, multi-institutional studies will be required to further define the biological mechanisms and clinical implications of this aggressive tumor subset.

## Supplementary Information

Below is the link to the electronic supplementary material.

Supplementary file1 (PDF 12577 KB)

## Data Availability

The presented data are available upon request.
