# Supplementary material for: Loss of meningothelial identity and mesenchymal fate switching in NF2-mutant meningiomas
Source: Acta Neuropathol. 2026 Apr 25;151(1):46. doi: 10.1007/s00401-026-03016-3 (PMC13110230; doi:10.1007/s00401-026-03016-3)
Supplement: Supplementary file 1 — Supplementary file1 (PDF 12577 KB) [file 401_2026_3016_MOESM1_ESM.pdf]

CASE 1

1-CNV plot of recurrent tumor

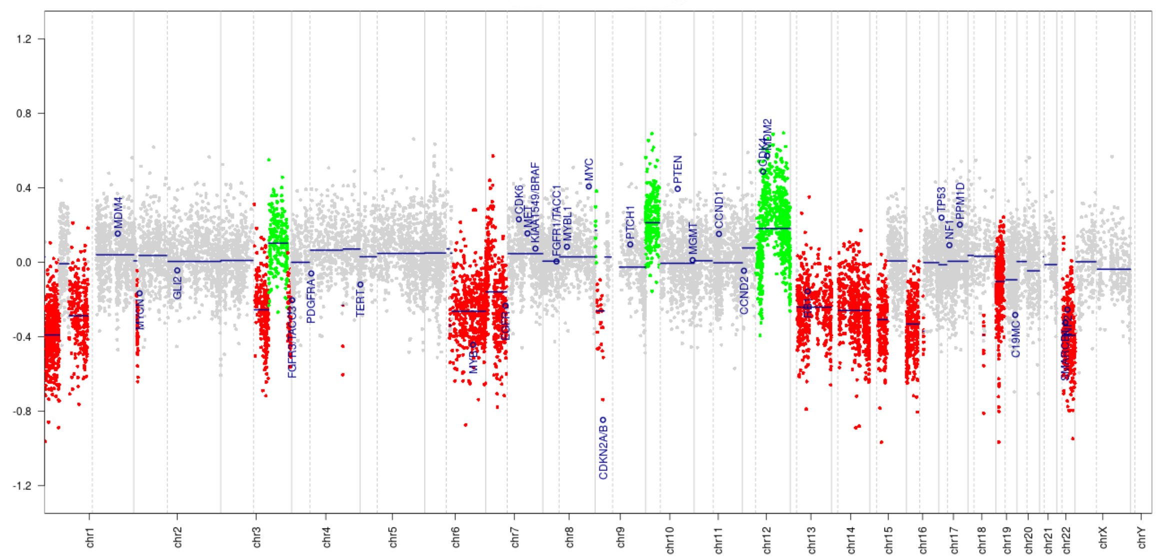

2-CNV plot of primary tumor

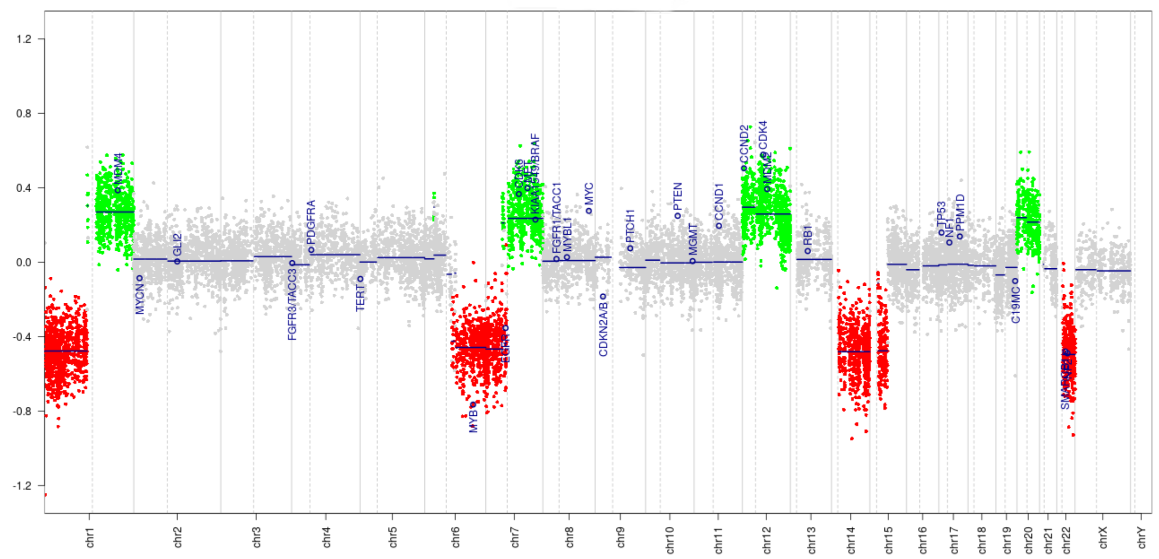

CASE 2

1-CNV plot of recurrent tumor

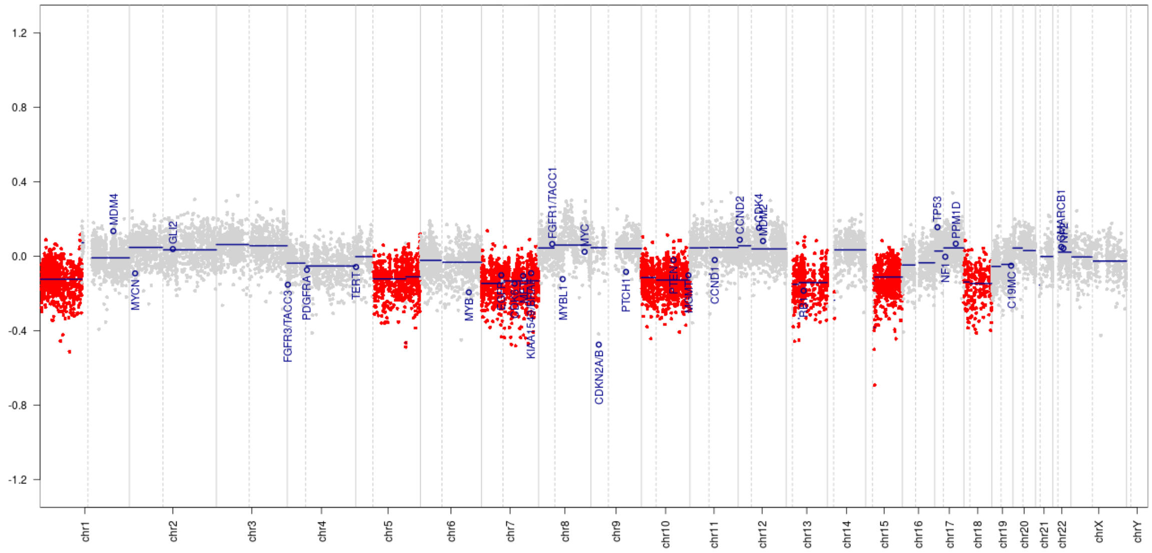

2-CNV plot of primary tumor

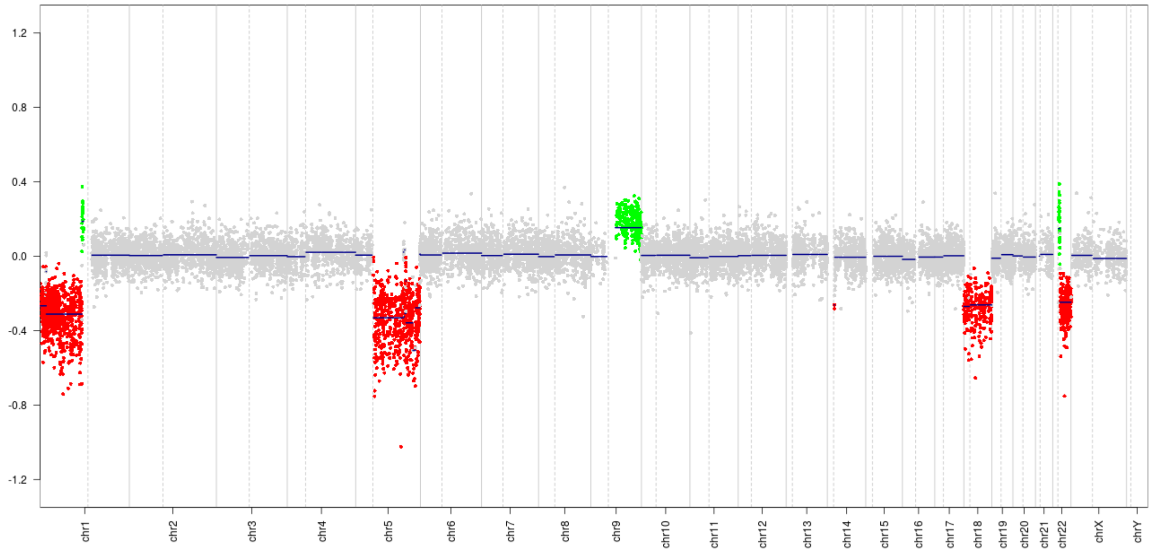

<u>CASE 3</u>

1-CNV plot of recurrent tumor

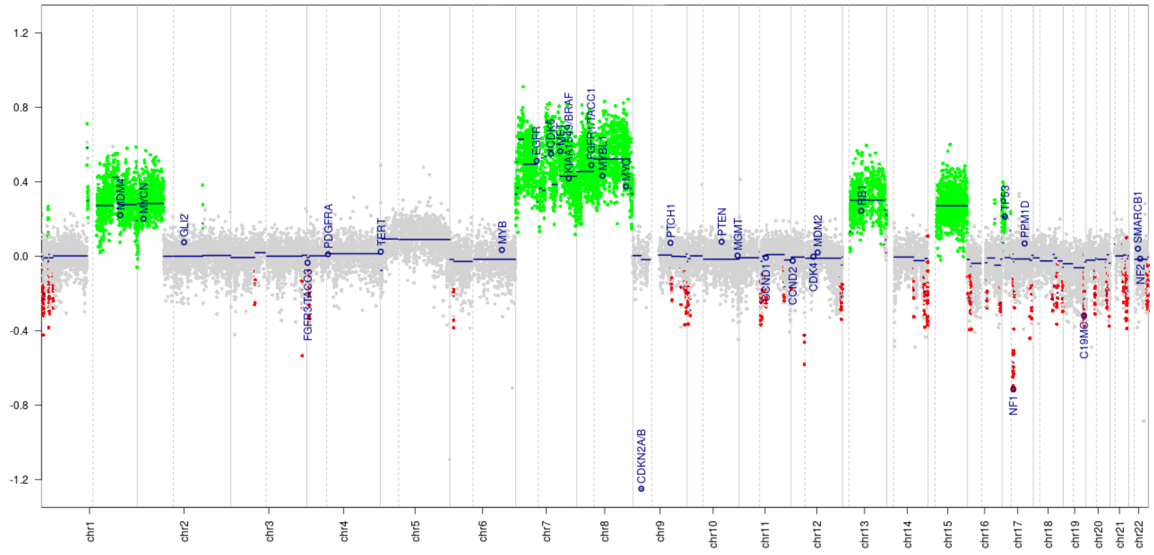

2-CNV plot of primary tumor

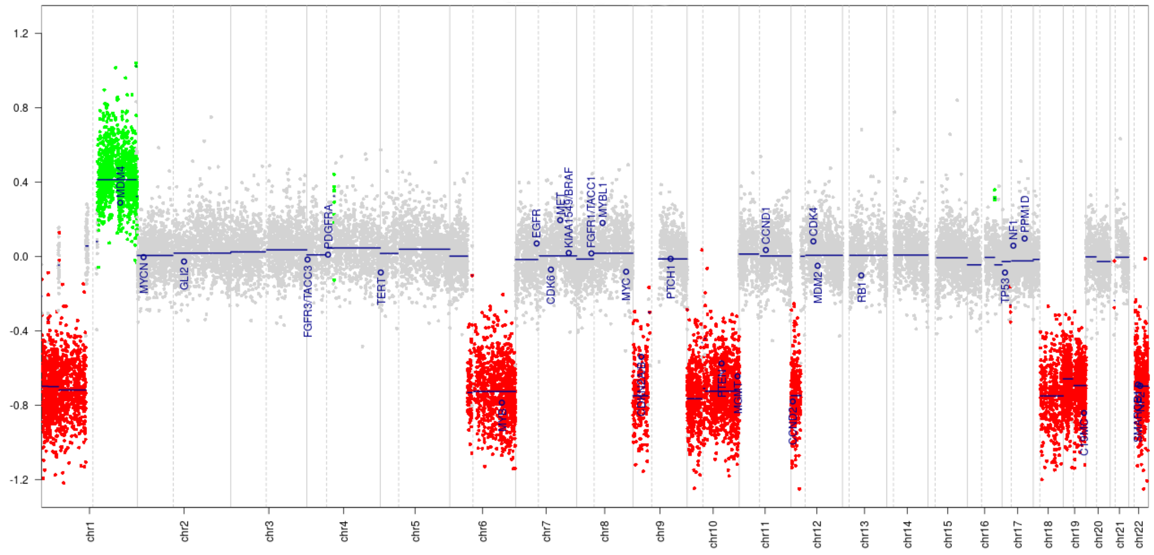

<u>CASE 4</u>

1-CNV plot of recurrent tumor

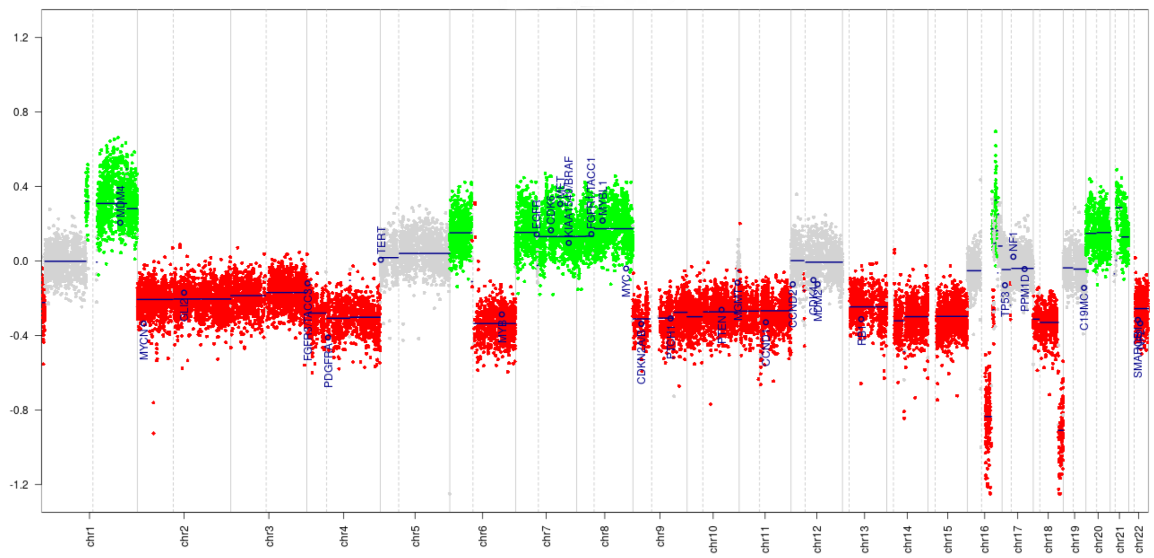

2-CNV plot of primary tumor

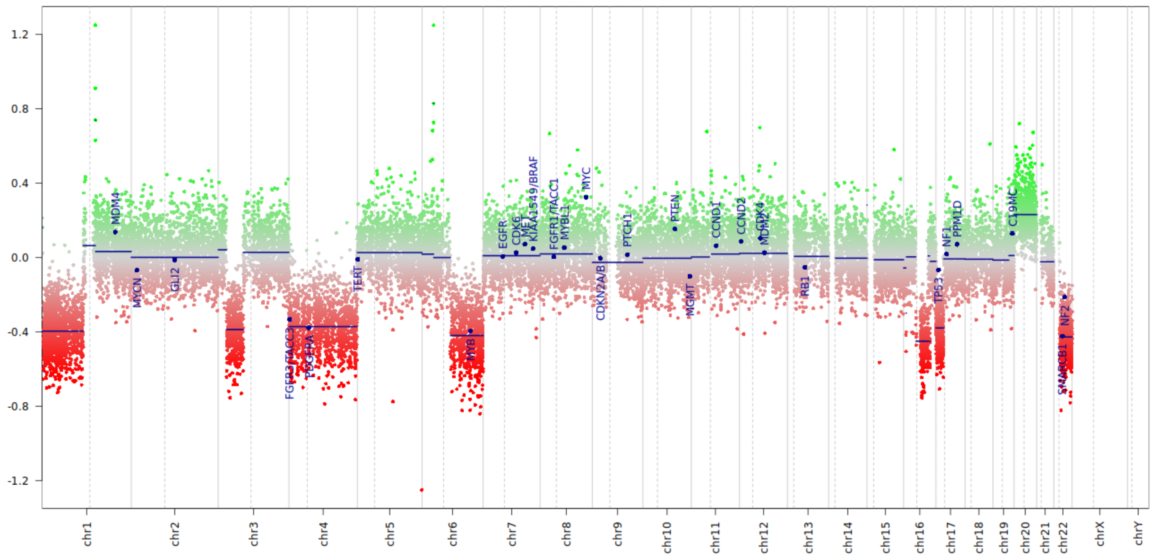

<u>CASE 5</u>

1-CNV plot of recurrent tumor

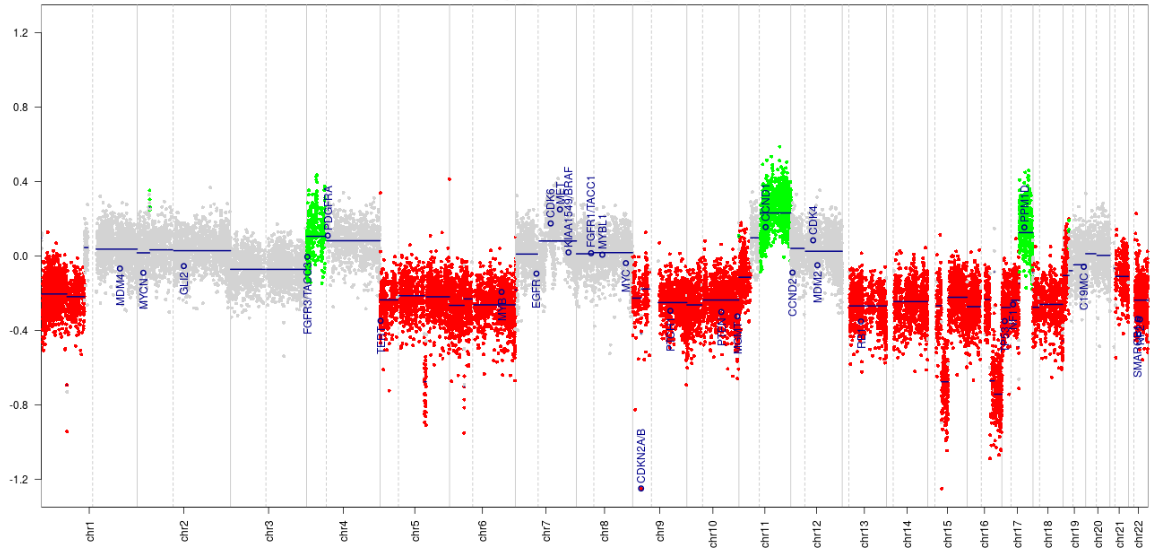

2-CNV plot of primary tumor

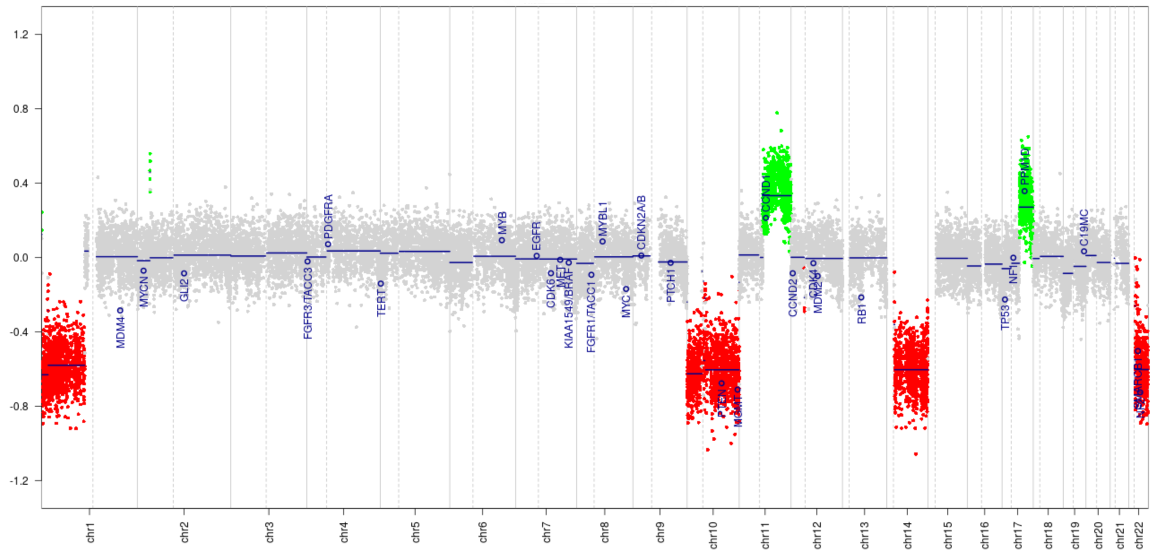

<u>CASE 6</u>

1-CNV plot of recurrent tumor

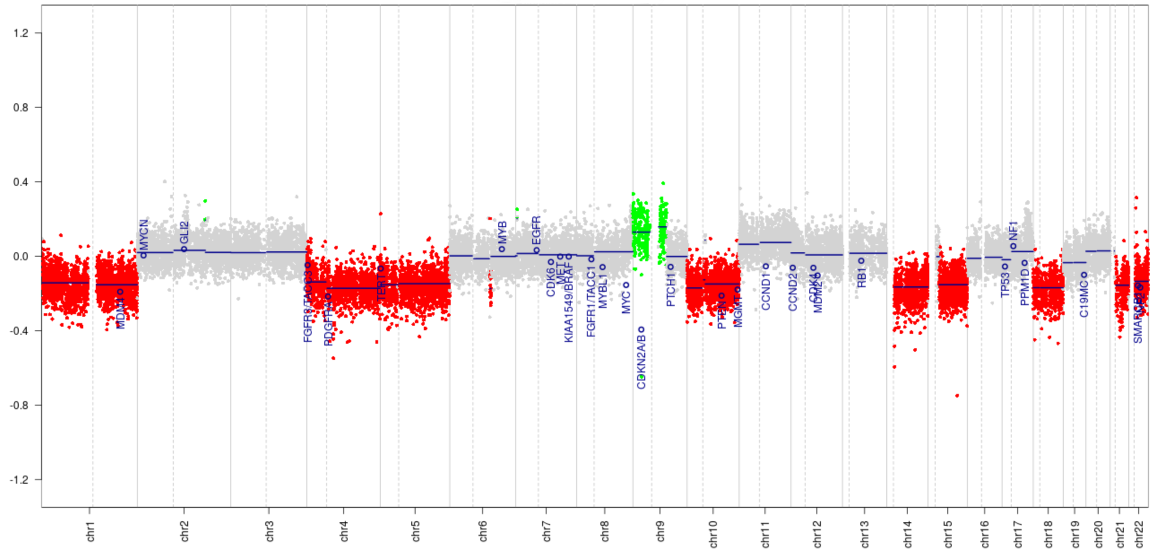

2-CNV plot of primary tumor

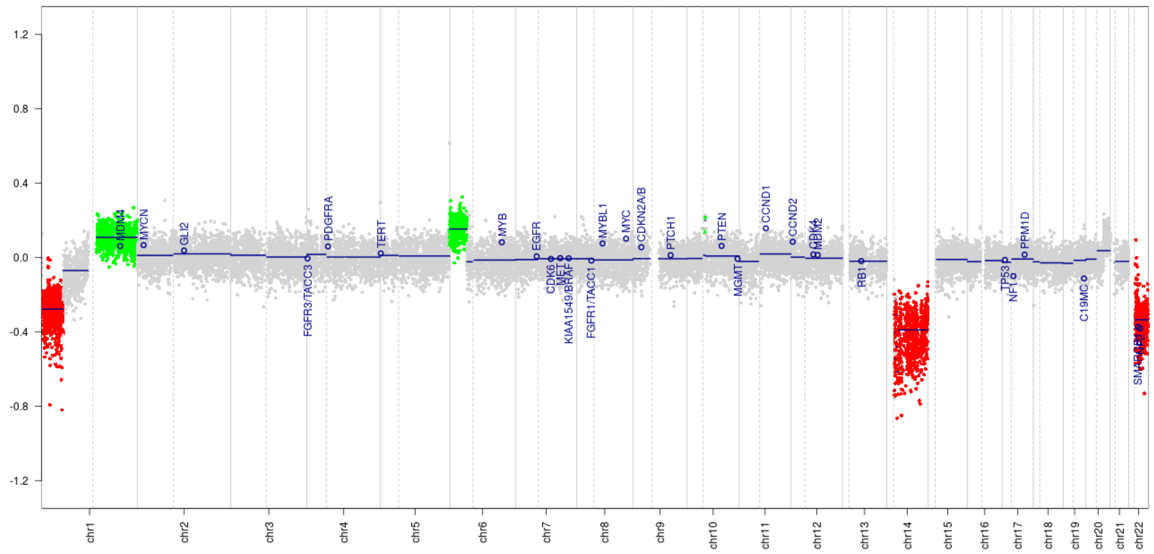

CASE 7

1-CNV plot of recurrent tumor

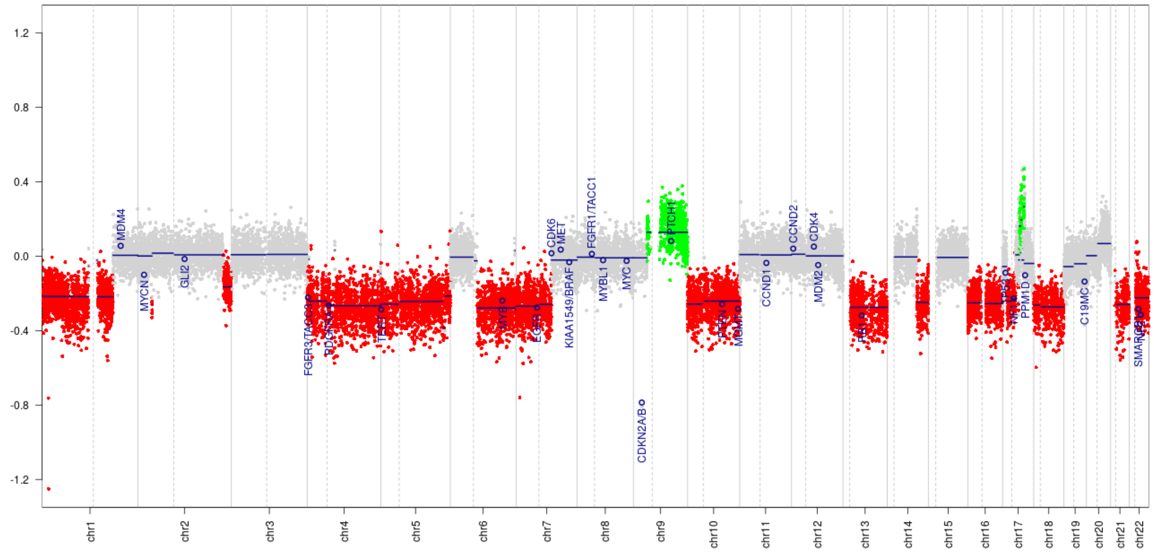

2-CNV plot of primary tumor

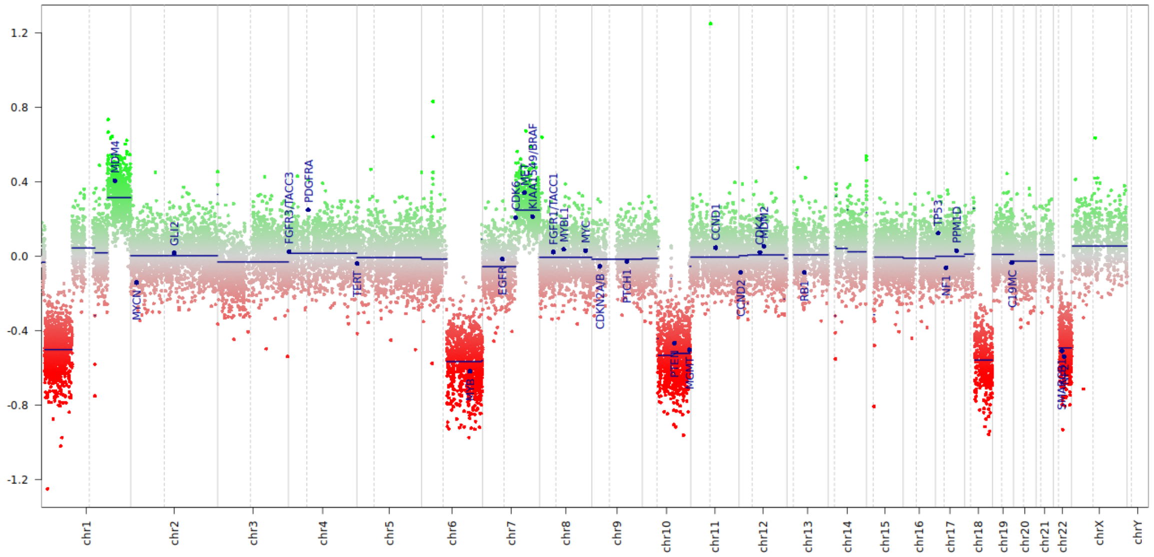

CASE 8

1-CNV plot of recurrent tumor

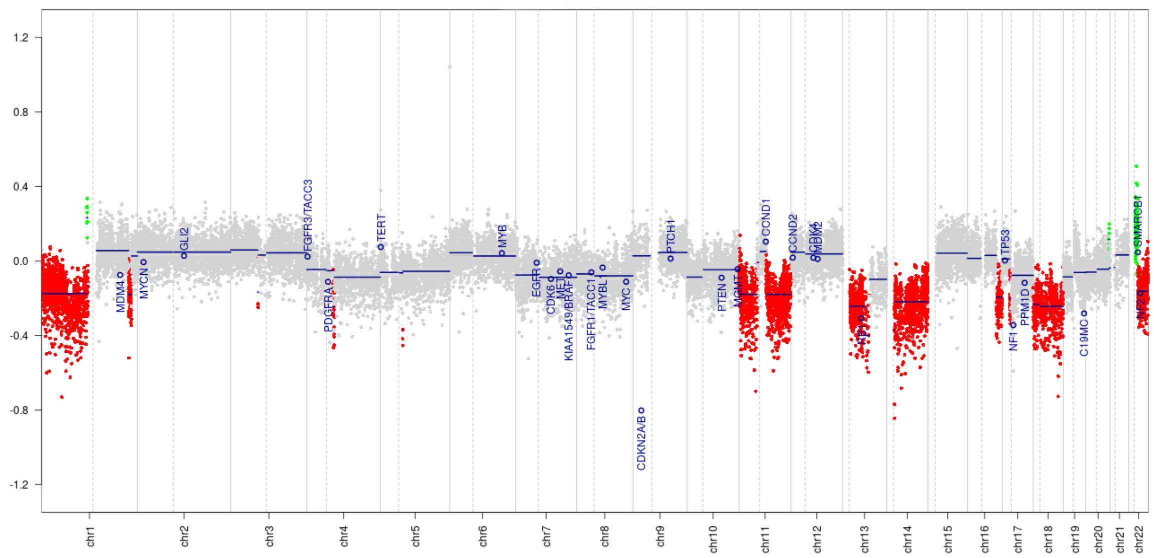

2-CNV plot of primary tumor

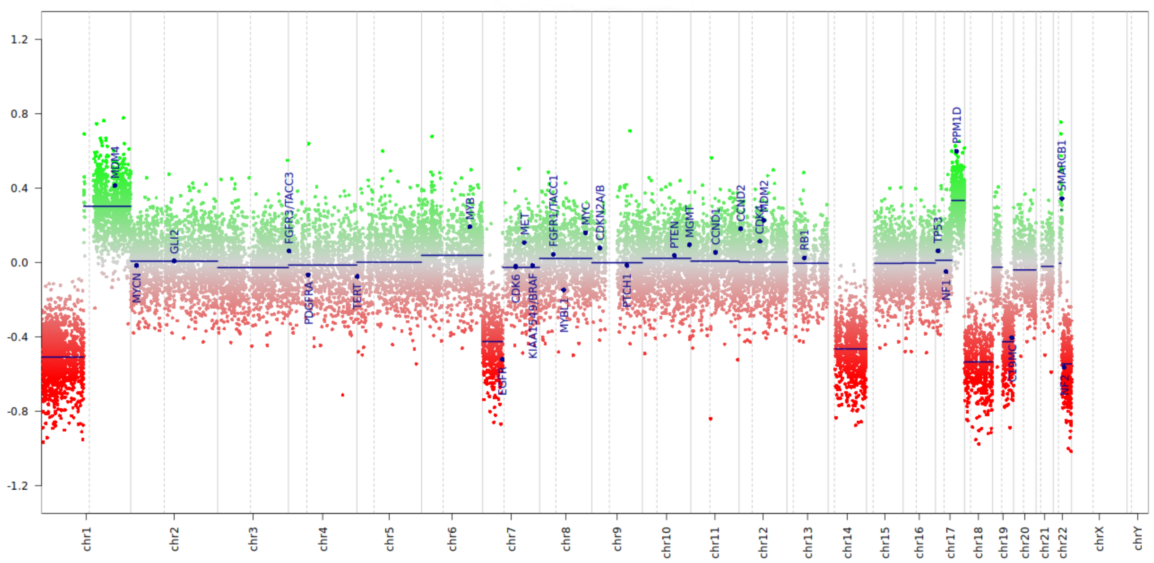

CASE 9

1-CNV plot of recurrent tumor

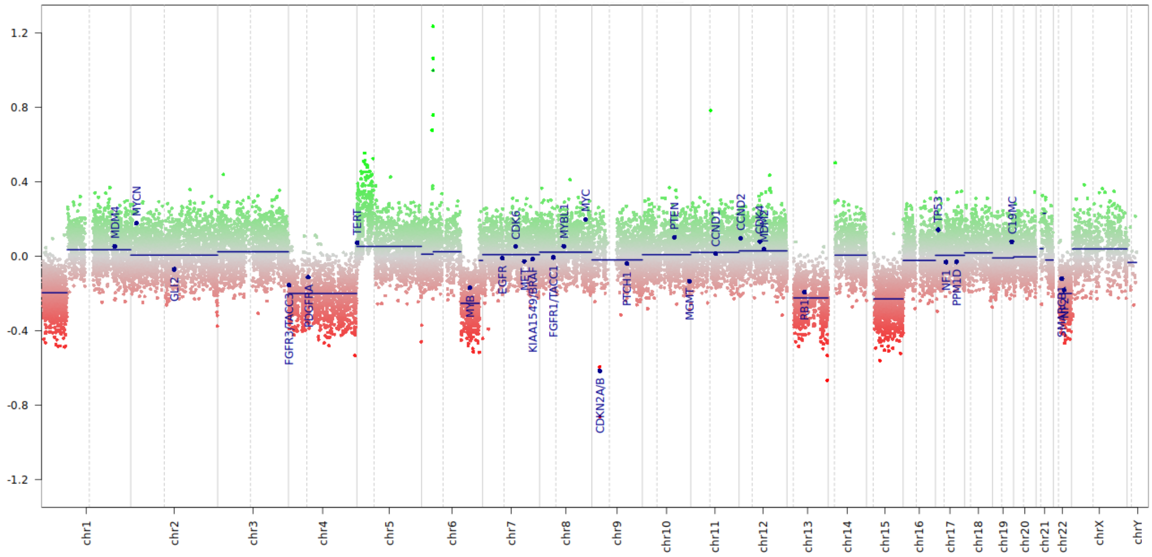

2-CNV plot of primary tumor

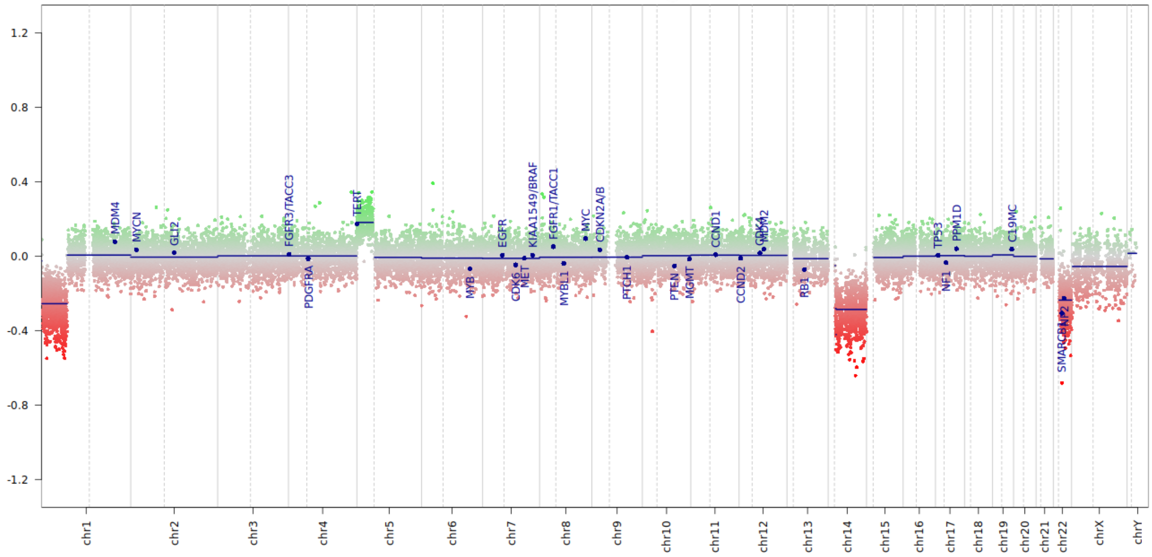

Suppl. Fig. 1 Comparison of copy number variation profiles between recurrent sarcomatous tumors and their primary meningioma counterparts demonstrating shared chromosomal alterations consistent with clonal relatedness.

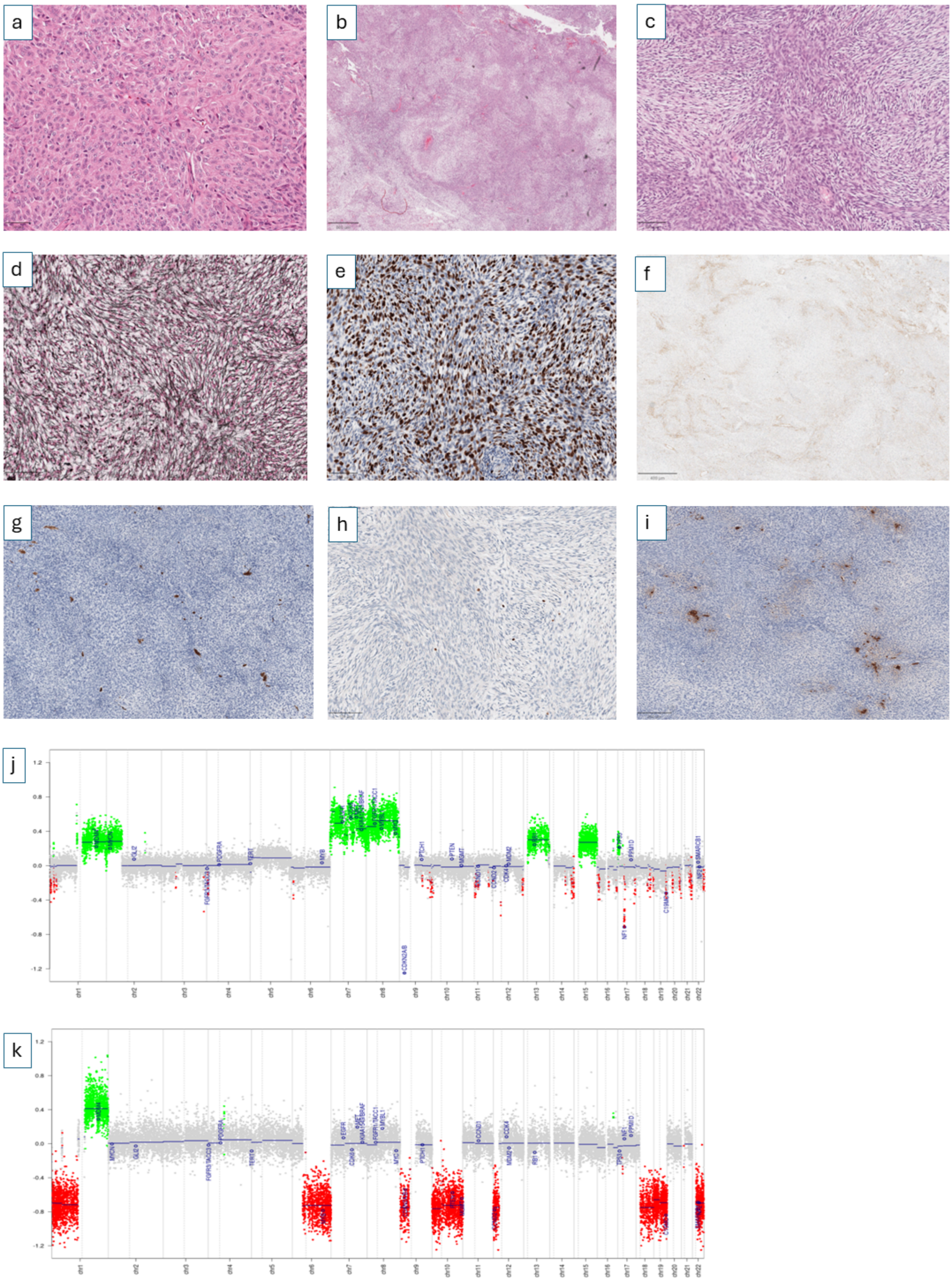

Suppl. Fig. 2 Sarcomatous recurrence of a WHO grade 2 meningioma without prior radiotherapy (#case 3). The primary tumor was diagnosed in February 2021 as a WHO grade 2 meningioma (a) and classified as MNG, int-B (score 0.99) using Brain Tumor Classifier v12.8. The recurrent tumor, resected in December 2021, showed spindle-shaped tumor cells arranged in interlacing fascicles with a storiform growth pattern and areas of alternating cellularity (marbling) (b, c). Additional histological features included necrosis, frequent mitoses, and dense reticulin networks (d). The proliferation index (Ki-67) was approximately 40% (e). Tumor cells showed patchy positivity for SSTR2A (f) and EMA (not shown). GFAP staining demonstrated extensive invasion into adjacent

CNS tissue with scattered GFAP-positive areas throughout the tumor (g). Tumor was predominantly negative for SOX10 (h) and showed patchy positivity for S100 (i). DNA methylation profiling classified the recurrent tumor as malignant peripheral nerve sheath tumor in Brain Tumor Classifier v12.8. Consistent with this classification, copy number analysis demonstrated homozygous deletion of NF1 and CDKN2A/B (m). Aside from shared copy number alterations such as gain of chromosome 1q (j, k), DNA sequencing from multiple tumor regions identified an identical NF2 nonsense mutation in both the primary and recurrent tumors.

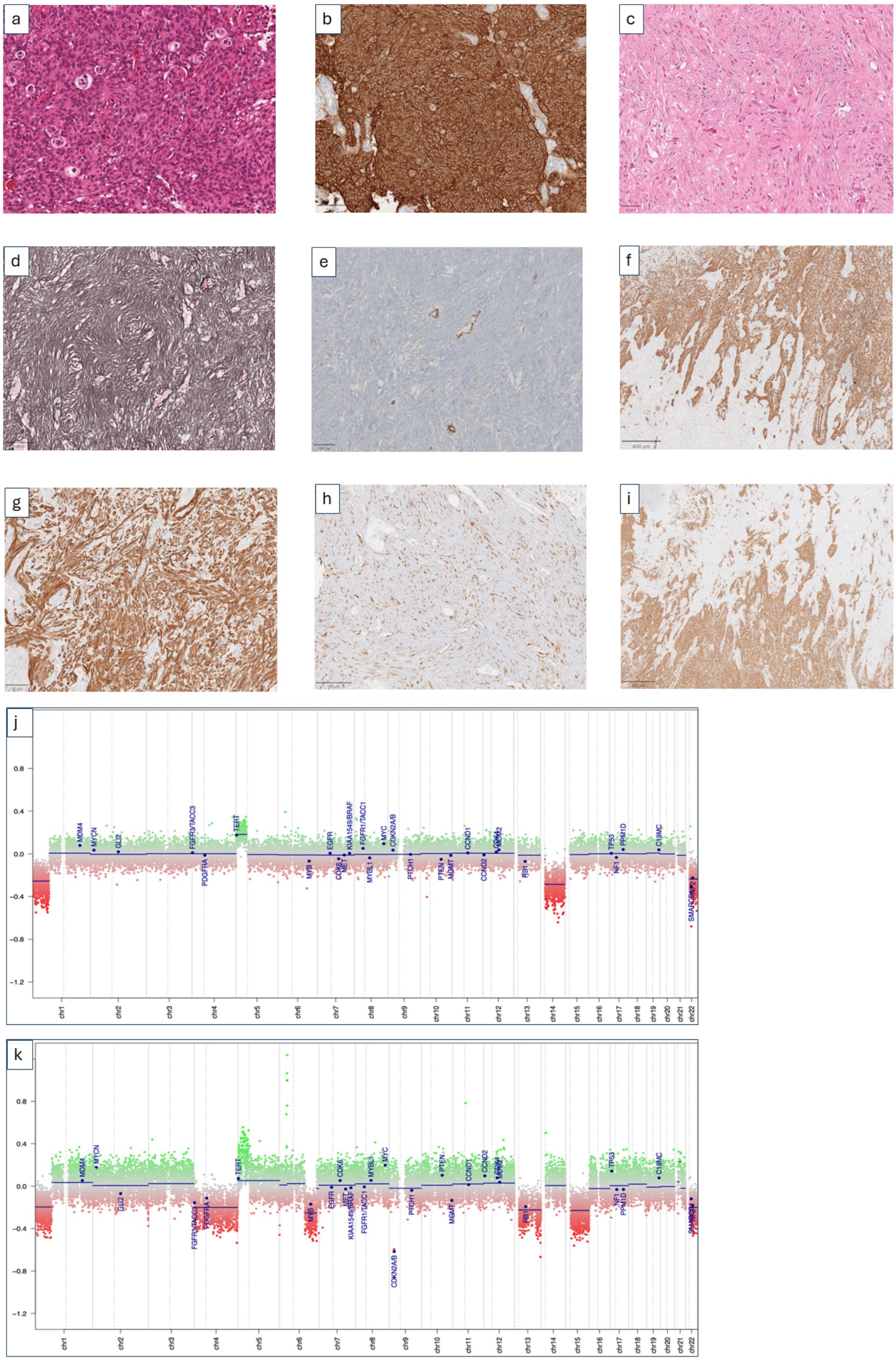

Suppl. Fig. 3 Sarcomatous transformation following radiotherapy in a recurrent meningioma (#case 9). The primary tumor, resected in March 2006, was diagnosed as a WHO grade 1 meningioma. The first recurrence in 2020 retained meningothelial features, including whorl formation (a) and SSTR2A positivity (b), and achieved the highest classification score for meningioma (0.86) using Brain Tumor Classifier v12.8. DNA sequencing revealed a TERT promoter mutation (c.-124C>T) and an NF2 frameshift mutation. The patient subsequently received postoperative radiotherapy. A second recurrence in May 2023 demonstrated sarcomatous morphology with dense reticulin fibers, necrosis, and brisk mitotic activity (c, d). Tumor cells lost expression of SSTR2A (e) and EMA but

showed strong positivity for SMA (f, g) and cytokeratin (h), particularly at the tumor–brain interface. Extensive invasion into adjacent CNS tissue was evident by GFAP staining (i). DNA methylation profiling of the recurrent tumor was unclassifiable using Brain Tumor Classifier v12.8 but showed the highest score (0.78) for undifferentiated sarcoma in Sarcoma Classifier v13.1. Copy number analysis demonstrated shared alterations between primary and recurrent tumors, including losses of chromosomes 1p and 22q and gain of chromosome 5p (j, k). DNA sequencing confirmed an identical NF2 frameshift deletion in both tumors.

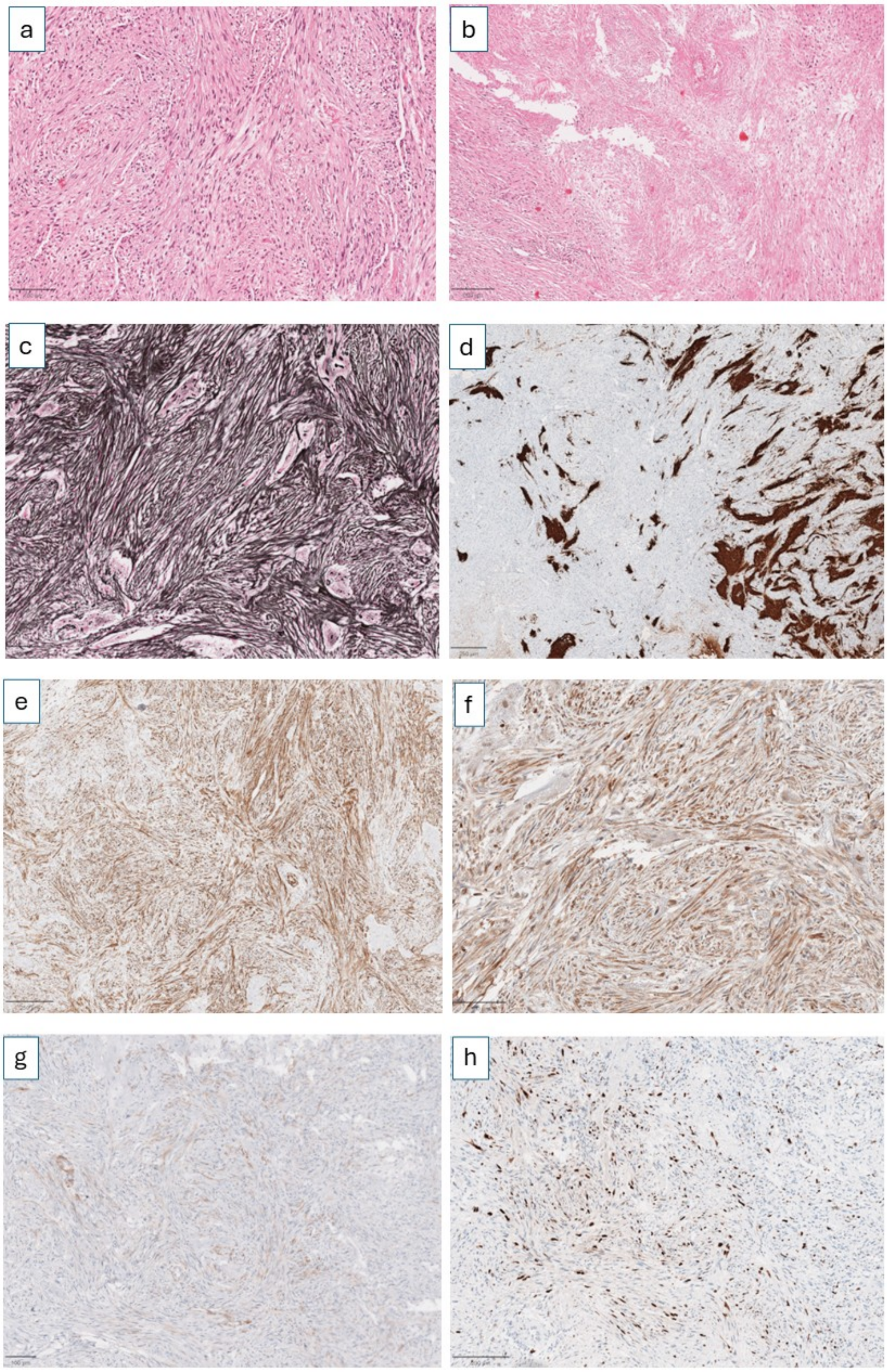

Suppl. Fig. 4 Intracranial sarcoma with inactivating NF2 mutation and focal meningothelial differentiation in the absence of a clinically recognized precursor meningioma (#case 10). The tumor, resected in April 2023, was initially diagnosed as a malignant spindle-cell tumor, not elsewhere classifiable. Histological examination showed sarcomatous morphology (a), areas of necrosis (b), and dense reticulin fibers (c). GFAP staining demonstrated extensive invasion into adjacent CNS tissue (d). Tumor cells showed strong expression of SMA (e) and SOX2 (f), with partial expression of the meningothelial marker SSTR2A (g). Ki-67 staining indicated high proliferative activity (h).

Suppl. Fig. 5 Results from Bethesda classifier v2 including copy number plots and classifier predictions based on DNA methylation profiles.

CASE 1

Summary | UMAP | Bethesda Classifier v2

Top hit classifiers results

	Best.match	Scores	Comment
Family	Mesenchymal_tumors	0.309	No match
Class	MPNST	0.848	No match

MGMT Status

Promoter	Status	pred
MGMT	Unmethylated	0.0827913614106033
		source: mgmstp27 R package

Copy Number Variation Profile

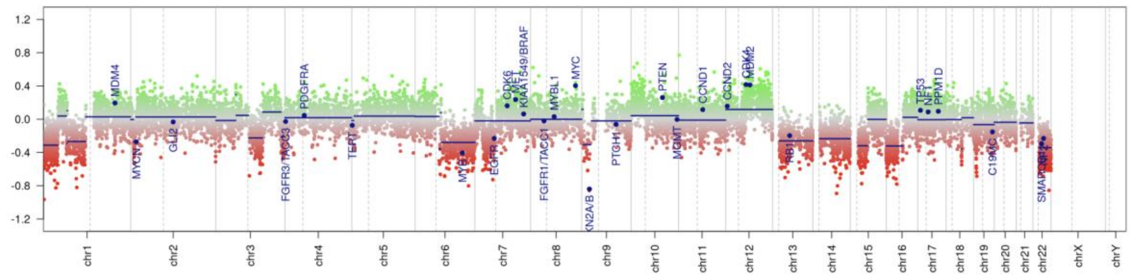

Regions	super_family	super_family_score	class	class_Score
ALL PROBES	Mesenchymal_tumors	0.28	MPNST	0.96
Gene_body	Mesenchymal_tumors	0.21	MPNST	0.92
**Shelf**	Epithelial_tumors	0.53	LUAD	0.67
Other_genomic_regions	Meningioma	0.16	MNG_INT_B	1.00
Shore	Mesenchymal_tumors	0.45	MPNST	0.93
OpenSea	Mesenchymal_tumors	0.44	ICMT_B	0.83
Island	Mesenchymal_tumors	0.41	MPNST	0.99
5'UTR	Mesenchymal_tumors	0.18	MPNST	0.58
Open_chromatin_probes	Mesenchymal_tumors	0.18	MPNST	0.72
TSS	Meningioma	0.42	MNG_INT_B	1.00

CASE 2

Summary | UMAP | Bethesda Classifier v2

Top hit classifiers results

	Best.match	Scores	Comment
Family	Epithelial_tumors	0.495	No match
Class	KIRC	0.407	No match

MGMT Status

Promoter	Status	pred
MGMT	Unmethylated	0.0230694562506741
		source: mgmstp27 R package

Copy Number Variation Profile

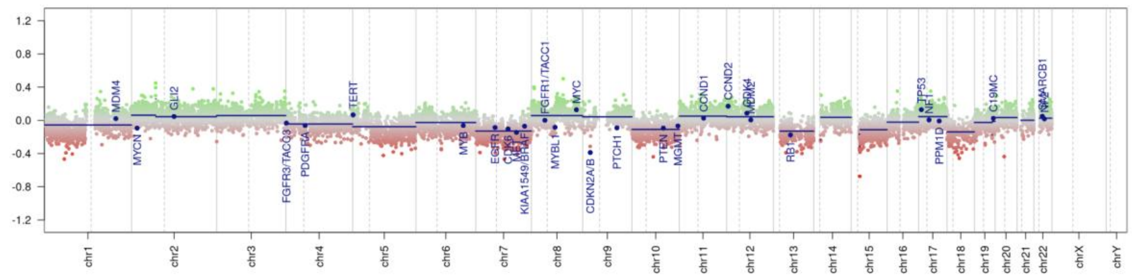

Regions	super_family	super_family_score	class	class_Score
ALL PROBES	Mesenchymal_tumors	0.25	ICMT_B	0.57
Gene_body	Epithelial_tumors	0.67	KIRC	0.33
Shelf	Epithelial_tumors	0.68	KIRC	0.38
Other_genomic_regions	Epithelial_tumors	0.46	BRCA_B	0.47
Shore	Epithelial_tumors	0.28	LUAD	0.37
OpenSea	Epithelial_tumors	0.51	BRCA_B	0.52
Island	Mesenchymal_tumors	0.31	ICMT_B	0.64
5'UTR	Epithelial_tumors	0.31	KIRC	0.50
Open_chromatin_probes	Epithelial_tumors	0.55	KIRC	0.42
TSS	Mesenchymal_tumors	0.25	ICMT_B	0.82

CASE 3

Summary | UMAP | Bethesda Classifier v2

Top hit classifiers results

	Best.match	Scores	Comment
Family	Mesenchymal_tumors	0.992	Matched
Class	ERMS	0.523	Suggested

MGMT Status

Promoter	Status	pred
MGMT	Unmethylated	0.00483244860459607
		source: mgmstp27 R package

Copy Number Variation Profile

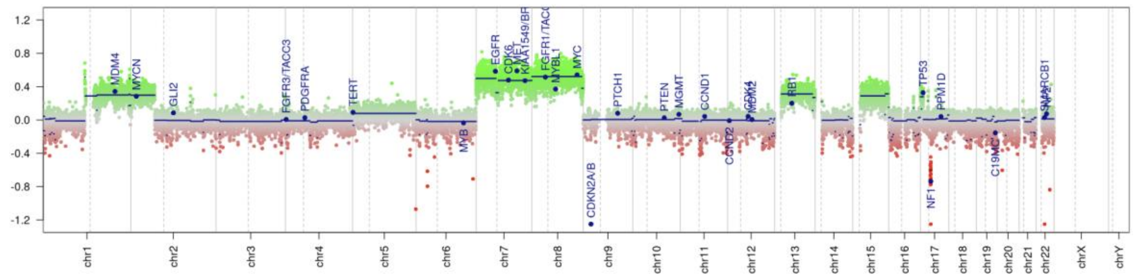

Regions	super_family	super_family_score	class	class_Score
ALL PROBES	Mesenchymal_tumors	0.98	MPNST	0.70
Gene_body	Mesenchymal_tumors	1.00	MPNST	0.88
Shelf	Mesenchymal_tumors	0.98	MPNST	0.65
Other_genomic_regions	Mesenchymal_tumors	1.00	ERMS	0.44
Shore	Mesenchymal_tumors	1.00	ERMS	0.64
OpenSea	Mesenchymal_tumors	0.98	MPNST	0.90
Island	Mesenchymal_tumors	0.98	ERMS	0.50
5'UTR	Mesenchymal_tumors	1.00	ERMS	0.48
Open_chromatin_probes	Mesenchymal_tumors	1.00	ERMS	0.39
TSS	Mesenchymal_tumors	1.00	ERMS	0.68

CASE 4

Summary | UMAP | Bethesda Classifier v2

Top hit classifiers results

	Best.match	Scores	Comment
Family	Mesenchymal_tumors	0.896	Suggested
Class	ERMS	0.766	Suggested

MGMT Status

Promoter	Status	pred
MGMT	Unmethylated	0.00856487739729843
		source: mgmstp27 R package

Copy Number Variation Profile

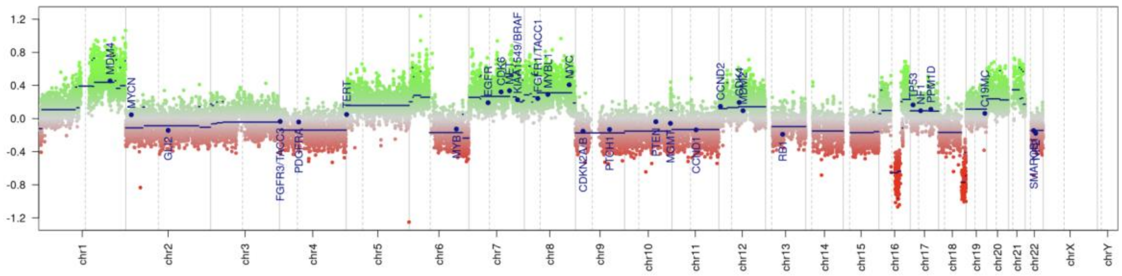

Regions	super_family	super_family_score	class	class_Score
ALL PROBES	Mesenchymal_tumors	0.85	ERMS	0.57
Gene_body	Mesenchymal_tumors	0.95	ERMS	0.82
Shelf	Mesenchymal_tumors	0.67	ERMS	0.97
Other_genomic_regions	Mesenchymal_tumors	0.98	ERMS	0.73
Shore	Mesenchymal_tumors	0.92	ERMS	0.82
OpenSea	Mesenchymal_tumors	0.93	ERMS	0.87
Island	SMARC_altered_tumors	0.53	ATRT_MYC	1.00
5'UTR	Mesenchymal_tumors	0.95	ERMS	0.91
Open_chromatin_probes	Mesenchymal_tumors	0.86	ERMS	0.64
TSS	Mesenchymal_tumors	0.95	ERMS	0.56

CASE 5

Summary | UMAP | Bethesda Classifier v2

Top hit classifiers results

	Best.match	Scores	Comment
Family	Glioblastoma	0.88	Suggested
Class	GBM_MES_ATYP	0.998	Suggested

MGMT Status

Promoter	Status	pred
MGMT	Methylated	0.817947323495158
		source: mgmstp27 R package

Copy Number Variation Profile

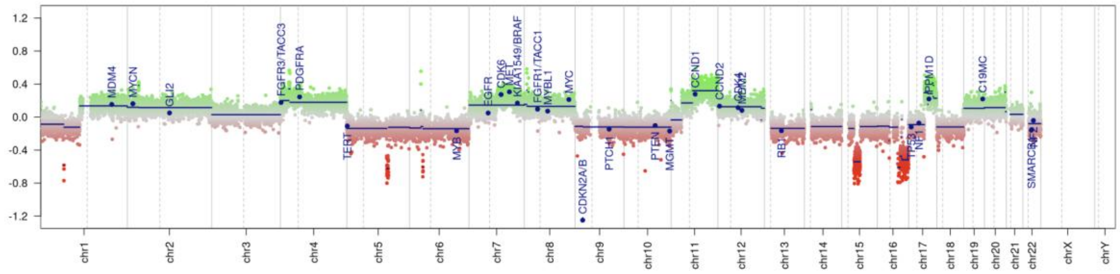

Regions	super_family	super_family_score	class	class_Score
ALL PROBES	Glioblastoma	0.42	GBM_MES_ATYP	1.00
**Gene_body**	Glioblastoma	0.96	GBM_MES_ATYP	1.00
**Shelf**	Glioblastoma	0.93	GBM_MES_ATYP	1.00
**Other_genomic_regions**	Glioblastoma	0.98	GBM_MES_ATYP	1.00
**Shore**	Glioblastoma	0.86	GBM_MES_ATYP	1.00
**OpenSea**	Glioblastoma	0.82	GBM_MES_ATYP	1.00
Island	Mesenchymal_tumors	0.31	MPNST	0.78
**5'UTR**	Glioblastoma	0.99	GBM_MES_ATYP	1.00
**Open_chromatin_probes**	Glioblastoma	0.97	GBM_MES_ATYP	1.00
**TSS**	Glioblastoma	0.98	GBM_MES_ATYP	1.00

CASE 6

Summary | UMAP | Bethesda Classifier v2

Top hit classifiers results

	Best.match	Scores	Comment
Family	Meningioma	0.757	Suggested
Class	MNG_BEN_3	0.565	Suggested

MGMT Status

Promoter	Status	pred
MGMT	Unmethylated	0.00264569289791412
		source: mgmstp27 R package

Copy Number Variation Profile

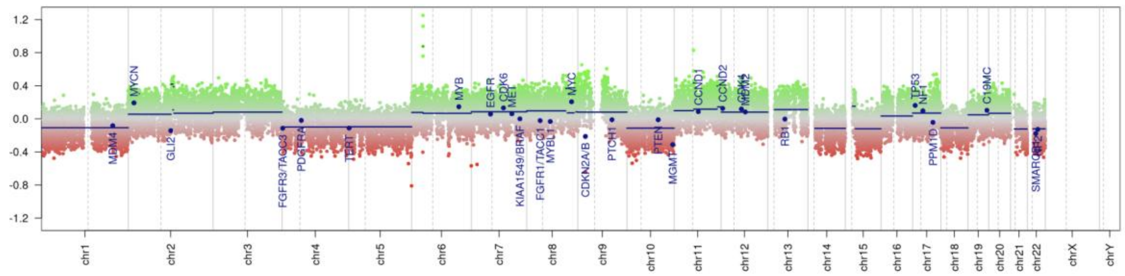

Regions	super_family	super_family_score	class	class_Score
**ALL PROBES**	Meningioma	0.83	MNG_INT_A	0.31
**Gene_body**	Meningioma	0.68	MNG_BEN_3	0.39
Shelf	Mesenchymal_tumors	0.47	ICMT_B	0.41
Other_genomic_regions	Glioblastoma	0.36	GBM_MES_ATYP	1.00
**Shore**	Meningioma	0.98	MNG_INT_A	0.57
OpenSea	Mesenchymal_tumors	0.38	ICMT_B	0.63
**Island**	Meningioma	0.97	MNG_INT_A	0.45
5'UTR	Meningioma	0.43	MNG_BEN_3	0.76
Open_chromatin_probes	Meningioma	0.50	MNG_BEN_3	0.55
**TSS**	Meningioma	0.90	MNG_MAL	0.55

CASE 7

Summary | UMAP | Bethesda Classifier v2

Top hit classifiers results

	Best.match	Scores	Comment
Family	Mesenchymal_tumors	0.263	No match
Class	ICMT_B	0.517	No match

MGMT Status

Promoter	Status	pred
MGMT	Unmethylated	0.0397328450918427
		source: mgmstp27 R package

Copy Number Variation Profile

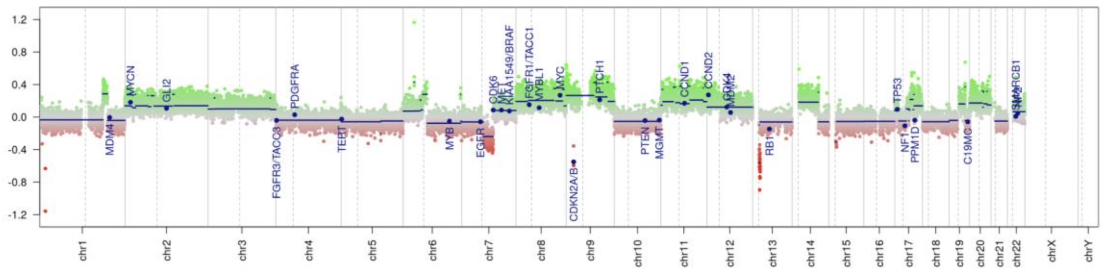

Regions	super_family	super_family_score	class	class_Score
ALL PROBES	Mesenchymal_tumors	0.28	ICMT_B	0.21
Gene_body	Mesenchymal_tumors	0.19	ICMT_B	0.31
Shelf	Epithelial_tumors	0.18	LUAD	0.44
Other_genomic_regions	Other_tumors	0.27	MELAN	0.98
Shore	Mesenchymal_tumors	0.37	CHORDM	0.29
OpenSea	Mesenchymal_tumors	0.23	ICMT_B	0.80
Island	Mesenchymal_tumors	0.38	MPNST	0.31
5'UTR	Glioblastoma	0.16	GBM_MES_ATYP	1.00
Open_chromatin_probes	Mesenchymal_tumors	0.20	ICMT_B	0.79
TSS	Mesenchymal_tumors	0.20	ICMT_B	0.48

CASE 8

Summary | UMAP | Bethesda Classifier v2

Top hit classifiers results

	Best.match	Scores	Comment
Family	Mesenchymal_tumors	0.729	Suggested
Class	CHORDM	0.352	No match

MGMT Status

Promoter	Status	pred
MGMT	Unmethylated	0.142054208670239
		source: mgmstp27 R package

Copy Number Variation Profile

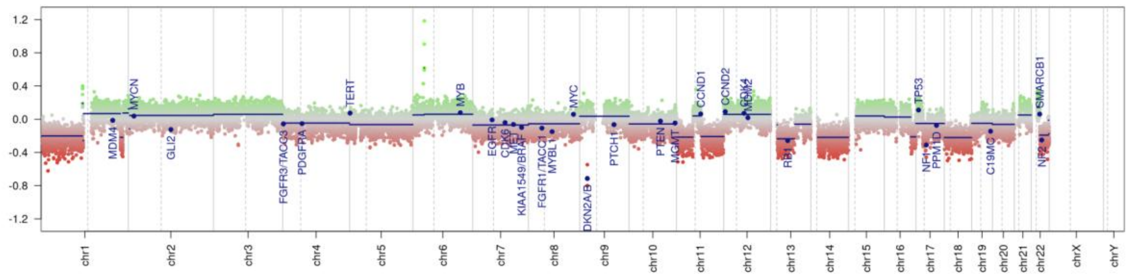

Regions	super_family	super_family_score	class	class_Score
**ALL PROBES**	Mesenchymal_tumors	0.59	CHORDM	0.21
**Gene_body**	Mesenchymal_tumors	0.88	CHORDM	0.29
**Shelf**	Mesenchymal_tumors	0.94	CHORDM	0.55
**Other_genomic_regions**	Mesenchymal_tumors	0.84	CHORDM	0.45
Shore	Mesenchymal_tumors	0.40	CHORDM	0.36
**OpenSea**	Mesenchymal_tumors	0.84	CHORDM	0.27
Island	Mesenchymal_tumors	0.39	CHORDM	0.18
**5'UTR**	Mesenchymal_tumors	0.93	CHORDM	0.47
**Open_chromatin_probes**	Mesenchymal_tumors	0.83	CHORDM	0.45
**TSS**	Mesenchymal_tumors	0.63	CHORDM	0.29

CASE 9

Summary | UMAP | Bethesda Classifier v2

Top hit classifiers results

	Best.match	Scores	Comment
Family	Mesenchymal_tumors	0.442	No match
Class	ICMT_B	0.356	No match

MGMT Status

Promoter	Status	pred
MGMT	Unmethylated	0.0495694873784454
		source: mgmstp27 R package

Copy Number Variation Profile

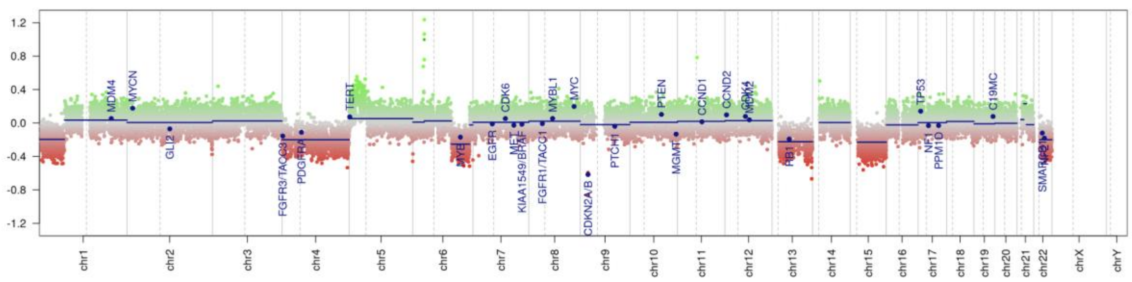

Regions	super_family	super_family_score	class	class_Score
ALL PROBES	Mesenchymal_tumors	0.43	ICMT_B	0.30
Gene_body	Mesenchymal_tumors	0.31	ICMT_B	0.30
Shelf	Glioblastoma	0.27	GBM_MES_ATYP	1.00
Other_genomic_regions	Glioblastoma	0.60	GBM_MES_ATYP	1.00
Shore	Mesenchymal_tumors	0.65	ICMT_B	0.26
OpenSea	Mesenchymal_tumors	0.30	ICMT_B	0.58
Island	Meningioma	0.90	MNG_INT_A	0.44
5'UTR	Glioblastoma	0.44	GBM_MES_ATYP	1.00
Open_chromatin_probes	Mesenchymal_tumors	0.44	ICMT_B	0.41
TSS	Mesenchymal_tumors	0.53	ICMT_B	0.29

CASE 10

Summary | UMAP | Bethesda Classifier v2

Top hit classifiers results

	Best.match	Scores	Comment
Family	Mesenchymal_tumors	0.983	Matched
Class	MPNST	0.181	No match

MGMT Status

Promoter	Status	pred
MGMT	Unmethylated	0.0418361844694283
		source: mgmstp27 R package

Copy Number Variation Profile

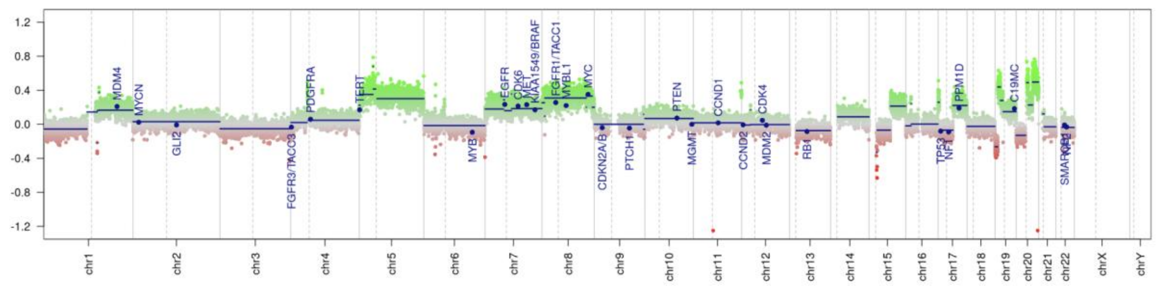

Regions	super_family	super_family_score	class	class_Score
**ALL PROBES**	Mesenchymal_tumors	0.98	SFT_HMPC	0.25
**Gene_body**	Mesenchymal_tumors	1.00	SFT_HMPC	0.17
**Shelf**	Mesenchymal_tumors	0.98	MPNST	0.19
**Other_genomic_regions**	Mesenchymal_tumors	0.99	MPNST	0.17
**Shore**	Mesenchymal_tumors	0.99	SFT_HMPC	0.21
**OpenSea**	Mesenchymal_tumors	0.98	MPNST	0.21
**Island**	Mesenchymal_tumors	0.94	ICMT_B	0.16
**5'UTR**	Mesenchymal_tumors	1.00	CHORDM	0.23
**Open_chromatin_probes**	Mesenchymal_tumors	0.99	MPNST	0.15
**TSS**	Mesenchymal_tumors	0.99	SFT_HMPC	0.15

CASE 11

Summary | UMAP | Bethesda Classifier v2

Top hit classifiers results

	Best.match	Scores	Comment
Family	Epithelial_tumors	0.426	No match
Class	LUAD	0.942	No match

MGMT Status

Promoter	Status	pred
MGMT	Unmethylated	0.0357711104230467
		source: mgmstp27 R package

Copy Number Variation Profile

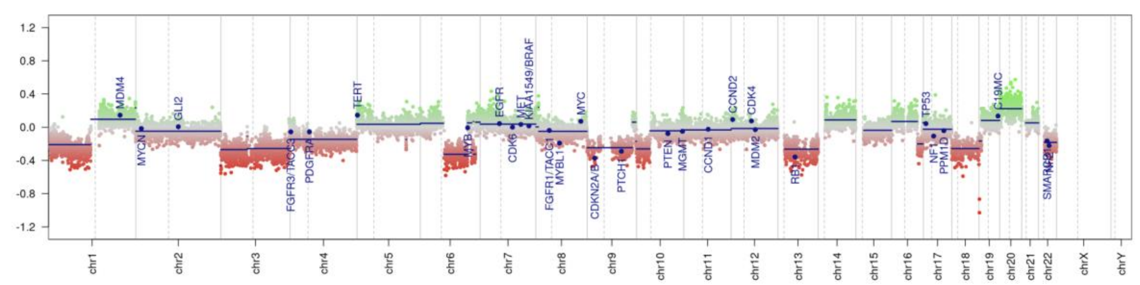

Regions	super_family	super_family_score	class	class_Score
ALL PROBES	Epithelial_tumors	0.22	LUAD	0.98
**Gene_body**	Epithelial_tumors	0.65	LUAD	0.97
Shelf	Epithelial_tumors	0.48	LUAD	0.98
Other_genomic_regions	Epithelial_tumors	0.26	LUAD	0.94
Shore	Epithelial_tumors	0.25	LUAD	0.94
**OpenSea**	Epithelial_tumors	0.53	LUAD	0.90
Island	Mesenchymal_tumors	0.33	MPNST	0.99
5'UTR	Epithelial_tumors	0.45	LUAD	0.96
**Open_chromatin_probes**	Epithelial_tumors	0.56	LUAD	0.87
TSS	Mesenchymal_tumors	0.23	MPNST	0.98
